# Defining Functions of Mannoproteins in *Saccharomyces cerevisiae* by High-Dimensional Morphological Phenotyping

**DOI:** 10.3390/jof7090769

**Published:** 2021-09-17

**Authors:** Farzan Ghanegolmohammadi, Hiroki Okada, Yaxuan Liu, Kaori Itto-Nakama, Shinsuke Ohnuki, Anna Savchenko, Erfei Bi, Satoshi Yoshida, Yoshikazu Ohya

**Affiliations:** 1 Department of Integrated Biosciences, Graduate School of Frontier Sciences, The University of Tokyo, Chiba 277-8562, Japan or farzang@mit.edu (F.G.); liuyaxuan_200@outlook.com (Y.L.); kaori.nakama-itto@edu.k.u-tokyo.ac.jp (K.I.-N.); ohnuki@edu.k.u-tokyo.ac.jp (S.O.); a.savchenko@maastrichtuniversity.nl (A.S.); 2 Department of Biological Engineering, Massachusetts Institute of Technology, Cambridge, MA 02139, USA; 3 Department of Cell and Developmental Biology, Perelman School of Medicine, University of Pennsylvania, Philadelphia, PA 19104, USA; okad@pennmedicine.upenn.edu (H.O.); ebi@pennmedicine.upenn.edu (E.B.); 4 Cardiovascular Research Institute Maastricht, Maastricht University Medical Center, ER 6229 Maastricht, The Netherlands; 5 School of International Liberal Studies, Nishi-Waseda Campus, Waseda University, Tokyo 169-8050, Japan; satosh@waseda.jp

**Keywords:** mannoprotein, cell wall, budding yeast, morphology, CalMorph

## Abstract

Mannoproteins are non-filamentous glycoproteins localized to the outermost layer of the yeast cell wall. The physiological roles of these structural components have not been completely elucidated due to the limited availability of appropriate tools. As the perturbation of mannoproteins may affect cell morphology, we investigated mannoprotein mutants in *Saccharomyces cerevisiae* via high-dimensional morphological phenotyping. The mannoprotein mutants were morphologically classified into seven groups using clustering analysis with Gaussian mixture modeling. The pleiotropic phenotypes of cluster I mutant cells (*ccw12*Δ) indicated that *CCW12* plays major roles in cell wall organization. Cluster II (*ccw14*Δ, *flo11*Δ, *srl1*Δ, and *tir3*Δ) mutants exhibited altered mother cell size and shape. Mutants of cluster III and IV exhibited no or very small morphological defects. Cluster V (*dse2*Δ, *egt2*Δ, and *sun4*Δ) consisted of endoglucanase mutants with cell separation defects due to incomplete septum digestion. The cluster VI mutant cells (*ecm33*Δ) exhibited perturbation of apical bud growth. Cluster VII mutant cells (*sag1*Δ) exhibited differences in cell size and actin organization. Biochemical assays further confirmed the observed morphological defects. Further investigations based on various omics data indicated that morphological phenotyping is a complementary tool that can help with gaining a deeper understanding of the functions of mannoproteins.

## 1. Introduction

The cell wall is a rigid structure that plays essential roles in establishing cell morphology and dictating the oval shape of budding yeast, *Saccharomyces cerevisiae*, and it also confers robustness on the cell by stabilizing internal osmotic conditions and serving as a site for cell wall enzymes to exert their effects [1,2,3]. Electron microscopic analysis has revealed that the yeast cell wall is a highly organized composite consisting of internal interconnected filamentous polysaccharides (1,3-β-glucan, 1,6-β-glucan, and chitin) and external non-filamentous glycoproteins (mannoproteins), which form a firm extracellular matrix similar to reinforced concrete [4,5]. Whereas 1,3-β-glucan is the major filamentous cell wall component [2,6] mainly responsible for dictating the yeast cell shape, non-filamentous mannoproteins, of which 36 members have been identified to date, have also been suggested to play fundamental roles in the cell wall [3].

Previous studies have indicated that individual deletions of genes encoding mannoproteins may result in subtle growth defects [7,8,9,10]. This can be partly explained by gene duplication, as 26 of 36 mannoprotein genes are duplicated [11]. Another reason is that cell wall defects caused by the lack of mannoproteins affect cell morphology rather than growth phenotypes [12]. Thus, the morphological phenotyping of mannoprotein mutants would provide more information on their functions, highlighting the importance of morphology as another metric with which to study the genes involved in cell wall assembly. In general, mannoproteins play a collective role in maintaining the cell wall structure [13], but differences in the localization, structure, and probably also the function of mannoproteins in the cell wall [3,8] suggest that the perturbation of individual genes may result in different morphological phenotypes [12]. Each mannoprotein is likely to have a distinct role in the cell wall, but the details have not been elucidated due to limited quantitative morphological analysis of mannoprotein mutants.

This study was performed to determine a responsibility assignment matrix (hereafter we call it responsibility matrix) through morphological clustering analysis of mannoproteins in relation to their molecular functions. For this purpose, high-dimensional morphological phenotyping was performed after extracting the morphological features of each mannoprotein mutant with the image processing program CalMorph [14]. Analyses of morphological abnormalities based on a powerful parametric approach revealed specific morphological phenotypes that will help with uncovering the responsibility matrix of mannoproteins in the yeast cell wall.

## 2. Materials and Methods

### 2.1. Strains and Growth Conditions

Of 36 mannoprotein gene-deletion mutants of the budding yeast, *S. cerevisiae* [3], we studied 32 mutants (Table S1) that were straightforward to observe as single cells under a microscope. These 32 mutants were isogenic derivatives of BY4741 (*MATa his3 leu2 met15 ura3*) and were purchased from EUROSCARF (Oberursel, Germany). Cells with mutations in the other four mannoprotein genes (*aga1*∆, *flo5*∆, *flo9*∆, and *dan4*∆) were not studied due to heavy cell aggregation. Mutant and wild-type (WT) strains were cultured under optimal growth conditions at 25 °C in nutrient-rich yeast extract peptone dextrose (YPD) medium containing 1% (*w*/*v*) Bacto yeast extract (BD Biosciences, San Jose, CA, USA), 2% (*w*/*v*) polypeptone (Wako Chemicals, Richmond, VA, USA), and 2% (*w*/*v*) dextrose, as described previously [14]. A WT diploid strain (BY4743) and the homozygous gene deletion mutants in the BY4743 background used for Western blotting were purchased from EUROSCARF (Oberursel, Germany).

### 2.2. Fluorescence Staining, Microscopy, and Image Processing

To minimize variation due to inconsistencies in data acquisition, we followed a precise protocol for the preparation, fixation, and fluorescence staining of yeast cells in the early *log* phase of growth (<5.0 × 10^6^ cells; 5 biological replicates), as described previously [15,16,17,18]. Briefly, yeast cells were fixed for 30 min in growth medium supplemented with formaldehyde (final concentration, 3.7%) and potassium phosphate buffer (100 mM, pH 6.5) at 25 °C. Yeast cells were then collected via centrifugation at room temperature and further incubated in potassium phosphate buffer containing 4% formaldehyde for 45 min. The fixed cells were subsequently prepared for fluorescence microscopy. First, actin staining was performed by treating the cells overnight with 15 U/mL rhodamine-phalloidin (Invitrogen, Carlsbad, CA, USA) and 1% Triton-X in phosphate-buffered saline (PBS). Second, cell wall mannoproteins were stained by treating cells for 10 min with 1 mg/mL fluorescein isothiocyanate-conjugated concanavalin A (Sigma-Aldrich, St. Louis, MO, USA) in P buffer (10 mM sodium phosphate and 150 mM NaCl, pH 7.2). Finally, after washing twice with P buffer, the yeast cells were mixed with mounting buffer (1 mg/mL *p*-phenylenediamine, 25 mM NaOH, 10% PBS, and 90% glycerol) containing 20 mg/mL 4′,6-diamidino-2-phenylindole (Sigma-Aldrich) to stain DNA.

Images of triple-stained cells were captured using an Axio Imager microscope equipped with a 6100 ECplan-Neofluar lens (Carl Zeiss, Oberkochen, Germany), a CoolSNAP HQ cooled charged coupled device (CCD) camera (Roper Scientific Photometrics, Tucson, AZ, USA), and AxioVision software ver. 4.5 (Carl Zeiss). The obtained images were quantified using CalMorph with regard to 501 morphological parameters related to the cell-cycle phase, actin cytoskeleton, cell wall, and nuclear DNA. The descriptions for each trait have been reported previously [14], and the CalMorph user manual is available at http://www.yeast.ib.k.u-tokyo.ac.jp/CalMorph/download.php?path=CalMorph-manual.pdf, accessed on 21 September 2019. Only those experiments containing at least 200 cells, detected by CalMorph, were considered for statistical analysis.

### 2.3. Data Analysis

All statistical analyses were performed using R (http://www.r-project.org, accessed on 21 September 2019). To assess the effects of genetic perturbation on the morphology of the mutants, we compared the cell morphological traits of the mutants with the corresponding WT distribution (i.e., null distribution) for each trait using an ANOVA model based on a generalized linear model (GLM). The GLM is an extension of the normal linear model in which predictors are linear but link functions are nonlinear to cope with violations of some standard assumptions of linear models [19]. These properties allow the analysis to cover probability distributions other than the Gaussian distribution. CalMorph generated 501 morphological parameters with which we established models based on the probability distributions for 490 unimodal parameters using the UNImodal MOrphological data pipeline (UNIMO; unpublished). Briefly, we first categorized CalMorph parameters into the following four data types: non-negative parameters, ratios, coefficients of variation (CVs; further converted to noise values, see below), and proportions. Then, we showed that these parameters could be explained well by 10 unimodal distributions to accommodate the statistical model used in the GLM: gamma, inverse gamma, and Weibull distributions for non-negative parameters; beta and logit-normal distributions for ratios; Gaussian, logistic, and reverse Gumbel distributions for noise parameters; and binomial and beta-binomial distributions for proportions. The best fit unimodal probability distribution for each parameter was eventually determined using the Akaike information criterion (AIC). CVs ($\frac{Population\;standard\;deviation\;\left(\sigma \right)}{Population\;mean\;\left(\mu \right)}$) are nonlinearly dependent on mean values [9]. We used LOESS (locally estimated scatterplot smoothing) regression with a smooth span (*f*) to uncouple this concomitant dependency. AIC values were used to choose the best-fitting model among various smooth spans (0.10 ≤ *f* ≤ 0.99). Finally, noise parameters were calculated as the residuals, i.e., observed value minus predicted value.

To estimate *Z*-values, once maximum-likelihood estimation converged, we transformed each morphological parameter to a *Z*-value via the Wald test (one-sample two-sided test) using the summary.gamlss R function [20]. The false discovery rate (FDR), the rate of type I error associated with rejecting the null hypothesis due to multiple comparisons, was estimated based on 2000 permutations.

### 2.4. Dimensionality Reduction and Clustering

To extract the most effective parameters, we performed principal component analysis (PCA), the most commonly used method for reducing dimensionality [21,22], on the obtained *Z*-values using the prcomp function (stats package). We then calculated the cumulative contribution ratio (CCR) to describe variation in the data. Based on the result, we used the first five principal components (CCR = 81.34%) for clustering analysis (Figure S1A).

Mixture model clustering is a probability-based approach in which we assume the dataset is best described as a mixture of probability models. In Gaussian mixture modeling (GMM), the most commonly used model-based clustering method [23], Gaussian distributions are fitted to the dataset. Gaussian distributions are randomly initialized and their parameters optimized iteratively to achieve a better fit. The expectation maximization algorithm estimates all parameters to assign members into *c* clusters. We employed the mclust package [23] to determine the underlying Gaussian mixture distributions (Figure S1B,C).

### 2.5. Kinetics of Cluster V Mannoproteins (Dse2 and Egt2)

#### 2.5.1. Yeast Media and Culture Conditions

Standard culture media and genetic techniques were used [24]. Yeast strains were grown routinely at 25 °C in synthetic complete (SC) minimal medium lacking specific amino acid(s) and/or uracil or YPD. Neutralized SC medium (pH 7.0) was used for live-cell imaging of green fluorescent protein (GFP) molecules exposed to the extracellular environment to prevent quenching of the GFP signal caused by the acidity of the standard SC medium.

#### 2.5.2. Constructions of Strains

New strains were constructed either by integrating a plasmid carrying a modified gene at a genomic locus or by transferring a deletion or tagged allele of a gene from a plasmid or from one strain to another via PCR amplification and yeast transformation; see footnotes in Table S2 [25,26,27].

#### 2.5.3. Primers and Plasmids

All PCR primers and plasmids used in this study are listed in Table S3. All PCR primers were purchased from Integrated DNA Technologies (Coralville, IA, USA). All new constructs were validated via sequencing performed at the DNA Sequencing Facility, University of Pennsylvania. The plasmids pFA6a-GFPEnvy-KanMX6, pFA6a-link-GFPEnvy-KanMX6, and pRS316-ENVY-FKS1(1-789) were described previously [28]. The plasmids bWL715 (pHIS3p:mRuby2-Tub1+3’UTR::HPH [29]) and pFA6a-URA3-KanMX6 [30] were generous gifts from Wei-Lih Lee (Dartmouth College) and John Pringle (Stanford University), respectively.

The following plasmids were generated for this study. To generate pFA6a-link-GFPEnvy-CaURA3, a ~0.7-kb *Pac*I-*Asc*I fragment containing *GFP^Envy^* from pFA6a-link-GFPEnvy-SpHis5 [27] was subcloned to replace the ~0.7-kb *Pac*I-*Asc*I region of pFA6a-link-yomApple-CaURA3 (#44879; Addgene, Watertown, MA, USA). To generate proHIS3-ymScarlet-I-TUB1-tTUB1-HPH (integrative, *hphMX*, expresses Tub1 N-terminally tagged with ymScarlet-I under the control of the *HIS3* promoter), two DNA fragments carrying either the ~0.7-kb ymScarlet-I insert or a ~6.5-kb plasmid backbone were amplified via PCR using the plasmid YIp128-proACT1-lifeact-ymScarlet-I-tADH1 (lab stock, integrative, *LEU2*, expresses Lifeact C-terminally tagged with ymScarlet-I under the control of the *ACT1* promoter) as the template DNA and the primers P1409 and P1412, or using the plasmid bWL715 as the template DNA and the primers P1410 and P1411, respectively. The resultant PCR products were then assembled using a Quick-Fusion cloning kit (Bimake, Houston, TX, USA). To generate pRS305-ENVY-FKS1(1-789), a ~4.2-kb DNA fragment carrying the partial open reading frame (ORF) of *GFP^Envy^-FKS1* (from ~1 kb of the *FKS1* promoter region, *GFP^Envy^*, and the *FKS1* ORF until residue 789 followed by a new stop codon) was amplified via PCR using pRS316-ENVY-FKS1(1-789) as the template DNA and the primers P222 and P512. The resultant PCR product was then subcloned into *Apa*I- and *Sac*I-digested pRS305 (integrative, *LEU2*) using a Quick-Fusion cloning kit.

#### 2.5.4. Imaging and Data Analysis

Time-lapse microscopy was conducted as described previously with slight modifications [31]. Cells were cultured to an exponential phase at 25 °C in SC medium, briefly sonicated at 15% power for 5 s to declump the cells (model Q55; Qsonica, Newtown, CT, USA), concentrated via centrifugation, and spotted onto concanavalin A-coated glass-bottom dishes. After a sufficient amount of cells had adhered to the bottom of each dish (> 50% cell cover in a microscopic field), the SC medium was replaced with neutralized SC liquid medium, and the dishes were then incubated at room temperature (23 °C) for 15 min to allow the cells to acclimatize. Images were acquired at room temperature with a spinning-disk confocal microscope (Eclipse Ti2-U; Nikon, Tokyo, Japan) with a 100× /1.49NA oil objective (CFI Apo TIRF 100×; Nikon) combined with a confocal scanner unit (CSU-X1; Yokogawa, Tokyo, Japan). An EMCCD camera (Evolve 512 Delta; Photometrics, Tucson, AZ, USA) was used for image capturing. Solid-state lasers for excitation (488 nm for GFP, and 561 nm for red fluorescent protein) were housed in a laser merge module (ILE-400; Spectral Applied Research, Richmond Hill, ON, Canada). The imaging system was controlled using MetaMorph (version 7.10.4.431, Molecular Devices, San Jose, CA, USA). Images were taken every 2 min with 11 z-stacks with a step size of 0.8 μm. Sum or maximum intensity projections were calculated using NIH ImageJ (1.51 h) [32]. To quantify fluorescence intensities, the integrated density at a division site was calculated from the sum intensity projection of an image stack by subtracting the fluorescence intensity in the background area from the total intensity in an ImageJ-drawn polygon covering the division site.

### 2.6. Biochemistry

Whole-cell protein extracts were prepared as described previously [33]. Briefly, cells were pelleted, treated with NaOH (0.1 N), and incubated on ice (5 min). Then, cells were pelleted, resuspended in SDS sample buffer including 62.5 mM Tris-HCl (pH 6.8), glycerol (10%), SDS (2%), β-mercaptoethanol (2%), and bromophenol blue (0.005%), boiled for 5 min, and pelleted. Afterward, the supernatants were loaded in a mini-gel (4–15%; Bio-Rad, CA, USA), and Western blotting was performed with rabbit anti–phospho-p42/44 MAPK (T202/Y204) antibody (Cell Signaling Technology, Danvers, MA, USA) and rabbit anti-yeast Rho1 antibody (Abmart, Berkeley Heights, NJ, USA). HRP-conjugated secondary antibodies were obtained from Millipore, and proteins were detected with an enhanced chemiluminescence system (ECL plus; Amersham, Darmstadt, Germany).

### 2.7. Similarity of Mannoprotein Mutants and Drug-Treated Wild-Type Cells in Morphology

Morphological profiles of ccw12∆ (I), ccw14∆ (II), cwp2∆ (IV), sun4∆ (V), ecm33∆ (VI), and sag1∆ (VII) were compared with WT cells treated with unicamycin, echinocandin B, nikkomycin Z, and hydroxyurea. Morphological data of the drug-treated cells were obtained from [34]. To investigate the profile similarity, first, CalMorph values were transformed to *Z*-values (Wald test) using the UNIMO pipeline (490 parameters). Then, the obtained *Z*-values of the WT replicates were exposed to PCA. Finally, *Z*-values of the mutants/drug-treated cells were projected onto PC axes of the WT. Pearson correlation coefficient (r) was calculated between each pair using first 94 PC scores (CCR = 99%).

### 2.8. Mannoprotein Analysis Based on Omics Studies

#### 2.8.1. Estimation of Fitness

To estimate the fitness of 32 mannoprotein mutants, a previously reported dataset containing the logarithmic strain growth rate coefficients of gene-deletion mutants grown on basal medium (LSC_basal_) was employed [10]. *p*-values were calculated to determine whether the fitness of each strain was significantly lower than that of the WT based on one tail of the estimated probability distribution, as described previously [12], using the pnorm function (stats package), and the results were corrected for family-wise error using the qvalue function in the qvalue package [35].

#### 2.8.2. Analysis of Protein Abundance and Protein–Protein Interactions

To determine the abundance of 32 mannoproteins at the protein level, mean values from 21 datasets were used as reported previously [36]. Protein–protein interaction (PPI) data were obtained from the BioGRID database [37]. We examined physical interactions (between interactors A and B) for each mutant of *S. cerevisiae* S288C (Taxonomy ID: 559292). Two types of PPI networks were considered: PPIs among the 32 mannoproteins and PPIs between each of the 32 mannoproteins and the whole proteome (i.e., the protein–protein interactome profile). Networks were visualized using Cytoscape 3.8.2 [38].

#### 2.8.3. Genetic Interaction Analysis

Genetic interaction (GI) data were collected as reported previously [39]. Significant interactions based on both queries and array analysis were considered for further analysis (*p* < 0.05). Two types of GI networks were considered: GIs among the 32 mannoprotein genes and GIs between each of the 32 mannoprotein genes and the whole genome (i.e., the genetic interactome profile). Finally, networks were visualized using Cytoscape 3.8.2 [38].

#### 2.8.4. Chemical-Genetic Profile Analysis

The chemical-genetic profiles of the 32 mannoproteins were obtained through text mining of the *Saccharomyces* Genome Database (SGD).

## 3. Results

### 3.1. Effects of Genetic Perturbations on Cell Morphology

We analyzed the morphology of mutants with deletions of individual genes encoding 32 mannoproteins using the image processing program CalMorph. To perform morphological phenotyping, it is necessary to consider the diversity in yeast morphological measurements. We applied different probability distribution models to accurately estimate the true value of each morphological parameter [17]. The use of 490 unimodal morphological parameters enabled a powerful approach, revealing biological information that may be masked with commonly used imaging methods. We found that cell morphology was remarkably altered: of 490 parameters, perturbations were detected in 136 parameters, consisting of 16, 77, and 43 parameters related to actin, cell, and nuclear DNA morphology, respectively (Wald test, FDR = 0.05; Table S4). This observation implies profound effects of mannoproteins on cell morphology, suggesting that mannoproteins may play roles in dictating cell shape and the progression of the cell cycle.

**Figure 1:**
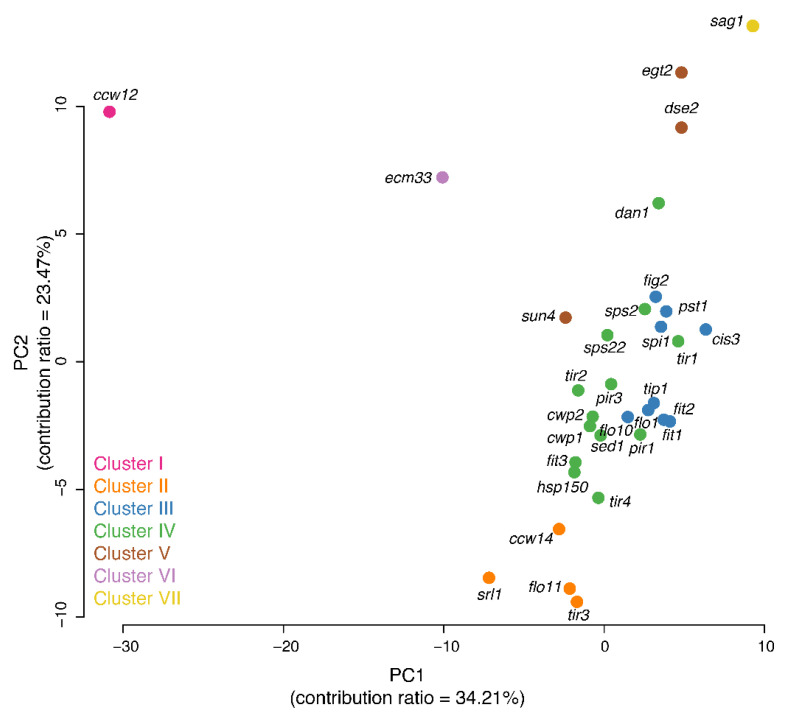
Two-dimensional principal component (PC) analysis score plot (biplot) illustrating clustering of the 32 mannoproteins. Each circle represents a single mannoprotein deletion mutant. The mixture likelihood values at individual points, based on the first five PC scores (CCR = 81.34%; Figure S1A) and a seven-component EEI model (Figure S1B), revealed data trends, including seven clusters. Mutants are color-coded.

To understand the morphological alterations more holistically, we reduced the number of dimensions of the morphological space to five via PCA of the Z-values of 136 significantly changed parameters; the first five principal components accounted for 81.34% of the variation (Figure S1A and Table S5). We then used GMM, one of the most commonly used model-based clustering methods for normally distributed data, to cluster the mannoprotein mutants (Figure S1B). The posterior probabilities associated with the data were evaluated in our GMM analysis to validate our clustering results (Figure S1C). Using GMM, we successfully clustered 32 mannoprotein mutants into seven groups (Figure 1). The *ccw12*Δ mutant, the single member of cluster I, was the mutant with the most abundant covalently linked cell wall protein. Members of cluster II (*ccw14*Δ, *flo11*Δ, *srl1*Δ, and *tir3*Δ) were mutants of serine-rich mannoproteins. Cluster III (nine mutants) and IV (13 mutants) accounted for more than half of the mannoprotein mutants, with their members exhibiting no or very small effects on cell morphology. Members of cluster V (*dse2*Δ, *egt2*Δ, and *sun4*Δ) were endoglucanase mutants. The *ecm33*Δ mutant in cluster VI had a mutation in a glycosylphosphatidylinositol (GPI)-anchored protein thought to be involved in bud morphogenesis. In the single member of cluster VII, the *sag1*Δ mutant, no morphological defects in vegetative growth had been reported previously.

### 3.2. Phenotype of the Cluster I Mutant (ccw12Δ)

**Figure 2:**
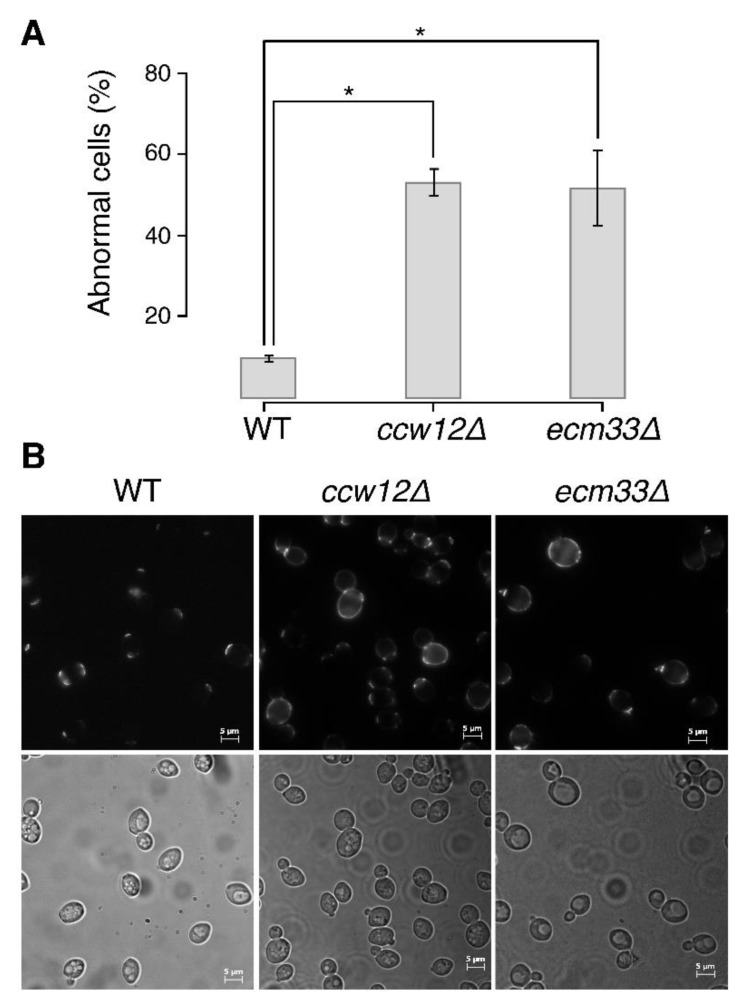
Abnormalities in *ccw12*Δ (cluster I) and *ecm33*Δ (cluster VI) cells. (**A**). Wild-type (WT) and mutant cells were grown in yeast extract peptone dextrose medium at 25 °C with shaking at 200 rpm until *log* phase. Cells (2.0 × 10^6^ cells) were suspended in 1 mL of phosphate-buffered saline (PBS) and mixed well with 5 μL of 5 mg/mL wheat germ agglutinin in PBS to stain chitin. After incubation at room temperature (30 min), the stained cells were washed three times and observed under a fluorescence microscope with a 4′,6-diamidino-2-phenylindole filter. The bar plot shows the percentages of abnormal *ccw12*Δ (cluster I) and *ecm33*Δ (cluster VI) cells in comparison with WT cells. Error bars indicate standard deviations. * *p* < 0.05 (*t* test). (**B**). Examples of chitin staining in WT, *ccw12*Δ (cluster I), and *ecm33*Δ (cluster VI) cells.

Among all mannoprotein mutants, *ccw12*Δ cells exhibited the greatest morphological alterations with 81 significantly changed parameters (Wald test, FDR = 0.05; Table S6). The *ccw12*Δ cells were larger in size at the S/G2 (C11-1_A1B and C101_A1B) and M phases (C11-1_C) and had a rounder cell shape (C115_A, C115_A1B, and C115_C) and wider neck at both the S/G2 (C109_A1B) and M phases (C109_C) (Figure S2). In addition, the *ccw12*Δ mutation affected bud morphogenesis, resulting in a rounded bud shape (C114_A1B and C114_C) and a disturbed budding direction (C106_A1B and C106_C) (Figure S3). Further phenotypic analysis using chitin staining revealed a significantly elevated population of cells exhibiting abnormal chitin staining (*p* < 0.05, *t*-test, Figure 2A,B), demonstrating that the loss of *CCW12* function had a detrimental impact on cell wall organization and assembly.

**Figure 3:**
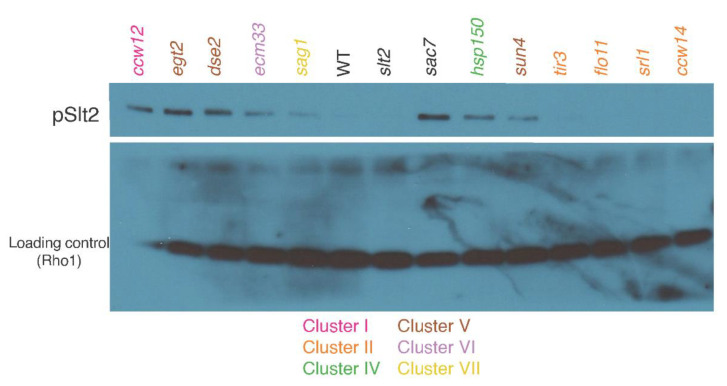
Western blotting of phosphorylated Slt2 (pSlt2, upper panel) and loading control Rho1 (lower panel). BY4743 (WT); *ccw12*Δ/*ccw12*Δ (cluster I); *ccw14*Δ/*ccw14*Δ, *flo11*Δ/*flo11*Δ, *srl1*Δ/*srl1*Δ, and *tir3*Δ/*tir3*Δ (cluster II); *hsp150*Δ/*hsp150*Δ (cluster IV); *dse2*Δ/*dse2*Δ, *egt2*Δ/*egt2*Δ, and *sun4*Δ/*sun4*Δ (cluster V); *ecm33*Δ/*ecm33*Δ (cluster VI); and *sag1*Δ/*sag1*Δ (cluster VII) cells were examined for the presence of phosphorylated Slt2. Rabbit antibody against phospho-p42/44 MAPK (T202/Y204) and rabbit antibody against yeast Rho1 were used to detect the phosphorylated Slt2 and Rho1, respectively. *slt2*Δ/*slt2*Δ and *sac7*Δ/*sac7*Δ were used as negative and positive controls, respectively, for phosphorylated Slt2. Mutants are color-coded according to Gaussian mixture model clustering of morphological data (see Figure 1).

Cell wall damage is accompanied by the activation of the cell wall integrity (CWI) pathway and the phosphorylation of Slt2 MAPK [40,41,42]. We found that the *ccw12*Δ mutant exhibited a marked increase in Slt2 phosphorylation, indicating that the cell wall was damaged in this mutant (Figure 3 and Figure S4).

Taken together, and given that Ccw12 is important for CWI, these observations indicate that this gene deletion causes pleiotropic defects in cell growth and morphology, possibly because of a severe loss of mannoprotein structures and functions.

### 3.3. Phenotype of Cluster II Mutants (ccw14Δ, srl1Δ, flo11Δ, and tir3Δ)

Cluster II mutants tended to produce larger mother cells at the M phase. The most noticeable morphological mutant in this cluster was *srl1*∆, which had a significantly larger mother cell size (C11-1_C), mother cell outline length (C12-1_C), and long axis (C103_C; Wald test, FDR = 0.05; Table S6). Both the mother cell outline length (C12-1_C) and long axis length (C103_C) of all cluster II mutants were larger than those in the other clusters, and nearly equivalent to those of the cluster I mutant (*ccw12*∆) (Figure S5). Therefore, we considered that the cluster II mutants exhibited perturbations in the mother cell size and shape at the M phase. There was no obvious increase in Slt2 phosphorylation, suggesting little cell wall damage in the cluster II mutants (Figure 3 and Figure S4).

### 3.4. Phenotype of Cluster V Mutants (dse2∆, egt2∆, and sun4∆)

Among the cluster V mutants, *egt2*∆ exhibited the greatest morphological changes, with significant differences in 31 parameters (Wald test, FDR = 0.05, Table S6). Morphological analysis of the cluster V mutants revealed common morphological features, such as the accumulation of cells at the M phase (D202 and D213) with actin patches localized at the bud neck (A109 and A118) (Figure S6). As actin patches are localized to the bud neck in cytokinesis, the morphological features of the cluster V mutants are suggestive of defects in cell separation. Consistent with this, cluster V genes (*DSE2, EGT2,* and *SUN4*) all encode cell wall mannoproteins similar to glucanase. It should be noted that the mutants exhibited no significant changes in bud cell size (C11-2_C and C12-2_C) or nuclear size (D14-2_C and D17-2_C) (Figure S7A), suggesting no defects in cell division but defects in physical attachment between mother and daughter cells. Mother cells frequently started the next budding cycle while still attached to old daughter cells (Figure S7B). The phosphorylation of Slt2 was increased in all cluster V mutants, suggesting cell wall damage (Figure 3 and Figure S4).

**Figure 4:**
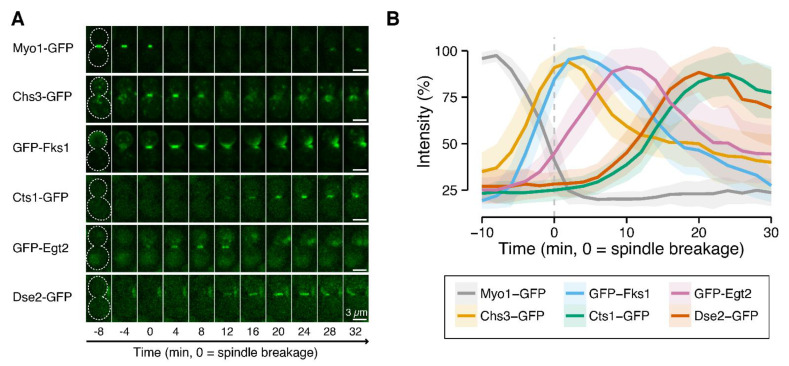
Kinetics of Dse2 and Egt2 (cluster V) proteins and proteins involved in cytokinesis and cell separation. (**A**). Images of green fluorescent protein (GFP)-tagged cluster V proteins and proteins involved in cytokinesis and cell separation. Montages of cells were created from frames selected from time-lapse series consisting of images taken at 2-min intervals. The white dotted line represents the cell outline. The following strains were used: YEF10861 (*MYO1-GFP mScarlet-TUB1*), YEF10856 (*CHS3-GFP mScarlet-TUB1*), YEF10857 (*GFP-FKS1 mScarlet-TUB1*), YEF10862 (*CTS1-GFP mScarlet-TUB1*), YEF10879 (*GFP-EGT2 mScarlet-TUB1*), and YEF10858 (*DSE2-GFP mScarlet-TUB1*). (**B**). Kinetics of the GFP-tagged proteins indicated in (**A**). The vertical dashed line shows timing of spindle breakage. Bold lines and associated shaded bands represent mean and SD values, respectively. *n* > 23 for each strain.

Glucanases are localized at the site of division in cytokinesis. To understand the precise timing of the function of glucanases in cell separation, we tagged cluster V gene products with GFP and performed quantitative time-lapse imaging to obtain information on real-time protein abundance at the division site (Figure 4) [28]. The accumulation peaks of both GFP-Egt2 and Dse2-GFP occurred after those of two secondary septum (SS)-forming enzymes, GFP-Fks1 and Chs3-GFP, suggesting that cluster V genes likely function after SS formation. Dse2-GFP exhibited accumulation kinetics remarkably similar to those of Cts1-GFP (*r* = 0.98, Table S7), a chitinase required for the degradation of the primary septum (PS) during cell separation. These observations imply that Dse2 may function in the same process as Cts1. Interestingly, the peak of GFP-Egt2 at the division site occurred between the peaks of the SS-forming enzymes and the peak of the PS-degrading enzyme, suggesting that Egt2 may be involved in cell wall remodeling or maturation, which is required for cell separation. Taken together, these results further support the involvement of cluster V genes in cell separation and explain the major cluster V mutant phenotype of mother cells with unseparated old daughter cells.

### 3.5. Phenotype of the Cluster VI Mutant (ecm33∆)

The *ecm33*∆ cells exhibited significant differences in 22 morphological parameters (Wald test, FDR = 0.05; Table S6) and were characterized by round mother cells (C115_A1B and C115_C), an altered neck position (C105_A1B and C105_C), and altered bud direction (C106_A1B and C106_C) (Figure S8). In addition, a reduced region of actin at the neck during the M phase (A9_C) and a lower proportion of cells exhibiting an isotropic pattern of actin (A117) were observed, suggesting that the defects in this mutant manifest before isotropic bud growth (Figure S8A). Consistent with these observations, the bud/mother cell size ratio (C118_C) (Figure S9) and ratio of cells with a large bud within budded cells (C125_C; Table S6) were both significantly decreased in *ecm33*∆. We observed a uniform distribution of chitin on the *ecm33*∆ cell surface (Figure 2B). The phosphorylation of Slt2 was increased in *ecm33*∆, suggesting cell wall damage in the cluster VI mutant (Figure 3 and Figure S4). These findings suggest possible roles of *ECM33* in bud growth and cell wall assembly.

### 3.6. Phenotype of the Cluster VII Mutant (sag1∆)

The *sag1*∆ cells exhibited significant differences in 28 morphological parameters (Wald test, FDR = 0.05). The sag1∆ mutation caused a smaller cell size at the G1 phase (C11-1_A, related to C103_A, C104_A, and C12-1_A) (Figure S10A and Table S6). Accordingly, the nucleus was also smaller at the G1 phase in *sag1*∆ cells (D102_A, D14-1_A, and D179_A) (Table S6). We observed the same trend (smaller bud size) at the M phase (C11-2_C, related to C107_C, C108_C, C12-2_C, C102_C, and C101_C) (Figure S10B and Table S6). Moreover, delocalized actin patches were observed frequently in *sag1*∆ cells (A111 and A112) (Figures S10C and S11), suggesting the perturbation of actin polarization and polarized bud growth. The size of the actin region in *sag1*∆ was more heterogeneous at the S/G2 phase (ACV7-1_A1B). We observed increased phosphorylation of Slt2 in *sag1*∆, suggesting cell wall damage in the cluster VII mutant (Figure 3 and Figure S4). Although *SAG1* is thought to play an important role only in the mating aggregation process [43,44], this is the first study revealing its effects on cell morphology during the vegetative growth phase.

### 3.7. Mannoprotein Gene Duplication

Many mannoprotein genes have been generated by gene duplication (Table S1 and Figure S12). Therefore, the effects of gene duplication were examined by measuring its impact on the morphological phenotype of each mutant (Table S8). More than 80% of the mutants with duplicated genes belonged to clusters III and IV and exhibited no obvious changes in their morphological phenotypes. The remaining *ccw12*∆ (I), *ecm33*∆ (VI), and *tir3*∆ (II) mutants exhibited changes in the morphological phenotype, but no obvious changes were observed in the deletion mutations of their counterparts. This is probably because gene duplication can result in functional bias. On the other hand, among strains with deleted genes unrelated to gene duplication, a significantly lower percentage of the mutants exhibited no obvious changes in the morphological phenotype (Table S8). Taken together, the duplication of mannoprotein genes resulted in a reduction in their functional effects, which made it difficult to examine the morphological phenotype of these gene-deletion strains.

### 3.8. Comparisons of Morphology and Fitness among Mannoprotein Mutants

**Figure 5:**
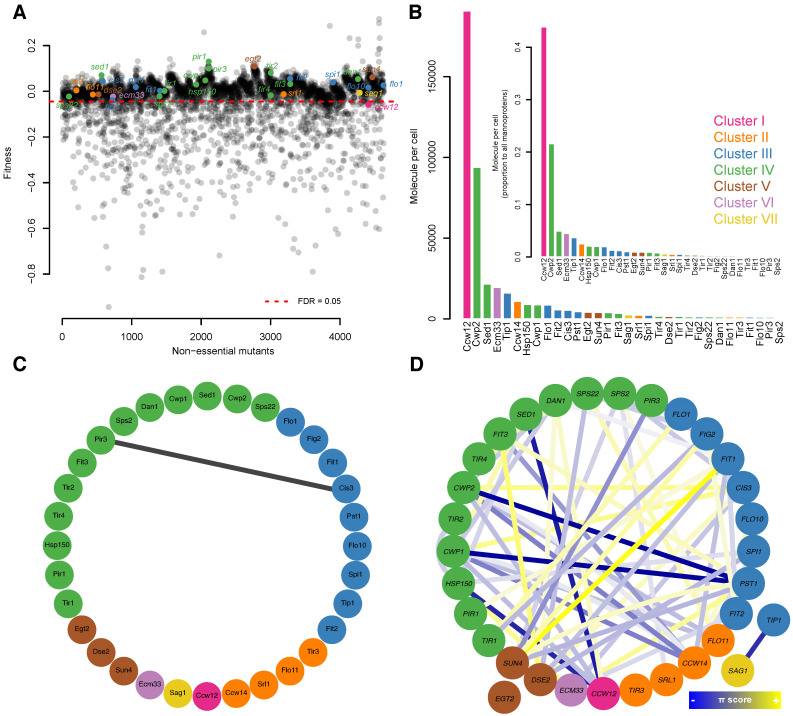
Mannoprotein analyses based on omics studies. (**A**). Scatter plot representing fitness-related defects. The dashed red line indicates a false discovery rate (FDR) of 5%. Data are from Warringer et al. [10]. Fitness data for *ccw14*Δ were not available in the dataset. (**B**). Bar plot showing the average cell wall mannoprotein abundances. Inset: A subset of the data. Data were obtained from [36]. (**C**). Protein–protein interactions among mannoprotein proteins are shown. Black line shows physical interaction. Data are from Oughtred et al. [37]. (**D**). Genetic interactions (GIs) among mannoprotein genes presented as blue (negative GI) or yellow (positive GI) lines (*p* < 0.05). Data are from Costanzo et al. [39]. *EGT2* did not have any significant GIs. In all sections, mutants are color-coded according to Gaussian mixture model clustering of morphological data (see Figure 1).

Associations between the comprehensive morphological phenotypes of the 32 mannoprotein mutants and the fitness of these mutants were assessed. Our morphological analysis including 490 morphological parameters revealed 12 mannoprotein mutants with significant abnormalities in at least one morphological parameter (Wald test, FDR = 0.05; Table S6). On the other hand, the fitness analysis of the gene-deleted strains revealed only one mutant (*ccw12*∆) with a significantly decreased growth rate in normal medium (Wald test, FDR = 0.05) (Figure 5A). The *ccw12*∆ mutant exhibited the greatest changes in its morphological phenotype. More differences were found in the morphological phenotype among the mutants than in fitness aspects, probably because of the high sensitivity of morphological phenotyping [12]. The morphological phenotype was also considered to be more greatly affected by the disruption of cell wall proteins.

### 3.9. Comparisons of Mannoprotein Mutants and Glycosylation-Defective Cells in Morphology

The remarkable differences in morphological phenotype found for *ccw12*∆ can be explained in terms of protein expression levels (Figure 5B). Yeast cells contain approximately 190,000 Ccw12 protein molecules per cell, accounting for more than 40% of all mannoproteins. The second most highly expressed mannoprotein is Cwp2, with approximately 93,000 molecules expressed per cell. As no morphological abnormalities were detected in *cwp2*∆, the expression level of a mannoprotein originally expressed at a high level would more markedly affect the morphology.

In order to know which cell wall metabolic pathways are relevant to mannoprotein function, we compare the morphology of *ccw12*∆ with those of the cells treated with cell wall agents. For this purpose, we used tunicamycin, echinocandin B, nikkomycin Z, and hydroxyurea, which affect protein glycosylation, 1,3-β-glucan synthesis, chitin synthesis, and DNA replication, respectively (Figure S13). We found that *ccw12*∆ is the most similar to the tunicamycin-treated cells (*r* = 0.813), implying a close relationship between protein glycosylation and mannoprotein function. *ccw12*∆ was also similar to the echinocandin B-treated cells (*r* = 0.723), but not similar to the cells treated with nikkomycin Z (*r* = 0.347) or hydroxyurea (*r* = 0.315). Taken together, these observations indicate that defects in *CCW12* resulted in serious damage to yeast cells, similar to defects in protein glycosylation and 1,3-β-glucan, which is the main filamentous component of the yeast cell wall.

### 3.10. Comparison of Morphological Clustering Results with Those from Analyses of Other Omics Data

We compared our clustering data with other omics data on interactions. A survey of comprehensive data on PPIs identified only one unidirectional interaction between Pir3 and Cis3 (Figure 5C). However, neither *pir3*∆ nor *cis3*∆ exhibited detectable changes in the morphological phenotype in the present study. Therefore, we could not infer the biological significance of the interaction between these two proteins based on morphological phenotyping. In addition, studying the PPI profile at the proteomic level did not reveal any similar patterns of PPI frequency among members of the same cluster (Figure S14A,B and Table S9). There were no associations between interactome profiles in each cluster either (Figure S14C).

With regard to GIs, we identified 26 positive and 42 negative relationships among the 32 mannoprotein genes. As with the PPI network, GIs among the 32 mannoprotein genes could not be directly linked to molecular functionality (Figure 5D). However, the lack of detectable morphological and fitness defects in many of the individual mannoprotein mutants may be explained by negative GIs comprising more than half (~61.7%) of all GIs. The lack of defects may be due to the existence of parallel pathways with the same or similar biological functions, such as the preservation of the cell wall structure. There were no noticeable GI patterns based on frequency of an interactome profile (Figure S15A,B and Table S10) or correlations between the members of a given cluster (Figure S15C).

Perturbations upon exposure to 106 different chemical compounds were tested in mannoprotein mutants, and the data are summarized in the SGD (Table S11). The chemical-genetic profiles of the mannoproteins were then visualized as a scatter plot in two-dimensional space representing the deletion mutants and chemicals (Figure S16). The comparison of the frequency of each mutant revealed that the chemical response phenotypes of *ccw12*∆ and *ecm33*∆ have been frequently tested. Of the seven clusters, only members of cluster V (*dse2*∆, *egt2*∆, and *sun4*∆) exhibited similar fitness defects with (S)-lactic acid (5.1% *w*/*v*) and miconazole (1000 μg/mL). Otherwise, the results of chemical-genetic profiling did not appear to be linked to molecular functionality.

**Figure 6:**
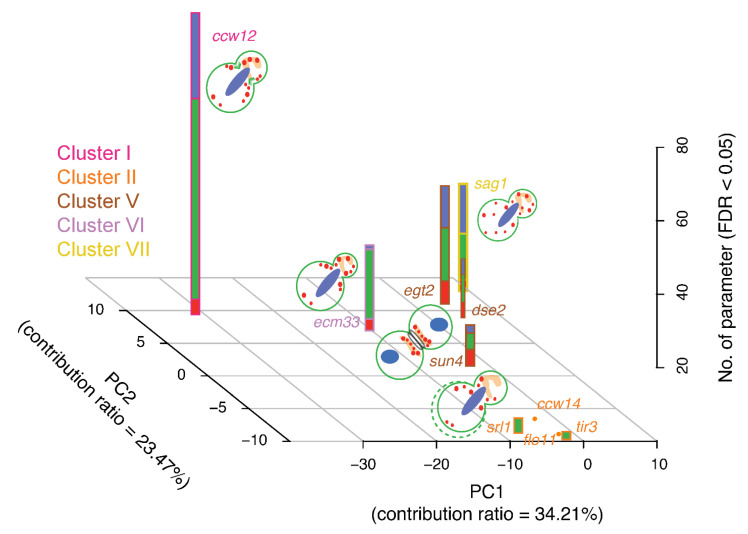
Schematic representation of the mannoprotein responsibility matrix. Stacked bars show the numbers of disturbed CalMorph parameters (Wald test, FDR = 0.05) related to actin, the cell wall, and the nucleus (illustrated in red, green, and blue, respectively) in mutants of clusters I, II, V, VI, and VII. Prominent implications of morphological defects caused by each mutation are illustrated in a small budding yeast cell where actin, the cell wall, and the nucleus are shown in red, green, and blue, respectively. Green dashed circle represents larger mother cell. Mutants are color-coded according to Gaussian mixture model clustering of morphological data (see Figure 1).

Morphological phenotyping of the mannoprotein mutants clearly accentuated unique aspects of the functional network that cannot be identified using other omics technologies. Thus, morphological phenotyping, as a complementary tool, provides deeper knowledge on cell wall organization, remodeling, and protein function. We succeeded in clustering 32 mannoproteins into seven groups based on their morphology and elucidated their specific functions in the cell (Figure 6).

## 4. Discussion

In this study, we used high-dimensional morphological phenotyping to gain a system-level understanding of 32 cell wall mannoproteins in *S. cerevisiae*. We found 12 mannoprotein mutants with significant abnormalities in at least one morphological parameter. Nearly 30% of the 490 unimodal morphological parameters examined were affected in the mannoprotein mutants, implying distinct roles of mannoproteins in cell morphology. Multivariate analysis revealed seven groups of mutants categorized according to the effects of the mutation on their functions. The results indicate that high-dimensional morphological phenotyping of mannoprotein mutants is an effective approach for determining the responsibility matrix of yeast mannoproteins, which is difficult to obtain with other omics technologies.

### 4.1. Ccw12 Is a Major Cell Wall Stabilizer

The highly pleiotropic morphological defects of *ccw12*Δ cells, including the wide neck, a typical phenotype of cell wall mutants [45], and altered cell shape for both mother and daughter compartments [46], clearly indicated the important role of Ccw12 as a major structural component of the cell wall [47]. This small (133 amino acid residues) and highly glycosylated GPI-anchored protein has been previously shown to impact the maintenance of newly synthesized areas of the cell wall [13] and cell fitness [10]. In addition, *ccw12*∆ has been reported to affect 473 genes acting in various cellular pathways, including 32 genes directly involved in the construction and remodeling of the cell wall [47]. Here, we confirmed that *ccw12*∆ cells exhibited the most significant morphological defects with differences found for 81 parameters. Ccw12 is localized at the presumptive budding site, around the bud, and at the septum [47], which explains the defect in the neck width of *ccw12*∆. An abnormal round cell morphology was also reported previously for *ccw12*Δ cells [13]. The defect in *CCW12* impacted another component of the cell wall because staining using wheat germ agglutinin (WGA) revealed the abnormal localization of chitin. Whereas chitin is located at the budding site in the WT strain, a uniform distribution of chitin on the cell surface was observed in *ccw12*Δ cells. Taken together, these results further confirmed that Ccw12 plays a major role in the maintenance of a rigid cell shape and the stabilization of the cell wall structure.

### 4.2. Cluster V Member Genes Encode Endoglucanases

After cytokinesis, mother and daughter cells undergo cell separation, which requires enzymatic digestion of the cell wall [48,49]. Dse2 is a well-known hydrolytic enzyme (glucanase) that functions exclusively in efficient cell separation from the daughter cell side [49,50]. Other cluster V member genes (*EGT2* and *SUN4*) have also been reported to encode glycosidases, and our results clearly showed that genetic perturbation prevented efficient daughter cell separation in all cluster V mutants. Consistent with the mutant phenotypes, co-localization studies have revealed that Dse2, Egt2, and Sun4 form a complex at the birth scar [51]. Cluster V mutants exhibited no defects in cell-cycle progression or daughter cell growth in the next cell cycle, indicating lesser effects of these genes in cell proliferation. Due to redundancy arising from intertwining pathways and the proteins involved, it was not clear how precisely diverse cell wall digestion systems are integrated to achieve effective cell separation; for example, *SUN4* genetically interacts negatively with some septin construction genes, including *CDC11* and *CDC12*, making its role in cell separation complex. Interestingly, our kinetic analysis revealed the temporal order among glucanases/chitinases. Dse2, Egt2, and Cts1 were deposited at the division site after septum synthesis was completed. However, Egt2 preceded Dse2 and Cts1. Therefore, Egt2 may be involved in cell wall maturation and making the wall architecture conducive for cell separation, whereas Dse2 and Cts1 are septum-hydrolyzing enzymes that arrive at the division site during the last step of cell separation. This finding suggests that glucanase- and chitinase-mediated cell separation is accomplished in a stepwise process. Consistent with the above observations, the expression of cell-separation genes is also regulated in a strict temporal order [52], as observed in our kinetic analysis. Early enzymes, such as Egt2, may function to remodel the cell wall or septum structure to facilitate the delivery of Cts1 to the PS [49].

### 4.3. ECM33 Plays a Role in Bud Growth

The molecular function of Ecm33 has not been fully elucidated. Previous studies suggested that it may play roles in determining cell shape [7], cell wall biogenesis [53,54], and apical growth [55]. Consistent with those previous reports, we confirmed that the roundness of mother cells (C115) and bud site selection (C106) were perturbed at both the S/G2 and M phases in *ecm33*∆. *ECM33* also has strong negative GIs with mannosyltransferase genes including *MNN11*, *ANP1*, and *HOC1*, which can explain the role of *ECM33* in cell wall assembly.

The smaller proportion of *ecm33*∆ cells exhibiting an isotropic pattern of actin suggests that *ECM33* functions before isotropic bud growth. However, apical bud growth seemed normal because the long and short axis lengths of the buds as well as their ratio were not significantly altered in *ecm33*∆ cells. Therefore, one possibility is that the apical and isotropic bud growth switch is delayed in the mutant. It has also been reported that *ECM33* deletion triggers the activation of the CWI pathway through the phosphorylation of Slt2 [54]. Although the CWI pathway is involved in cell-cycle checkpoints, such as the cell wall integrity checkpoint and cell morphological checkpoint, it is unlikely that any cell-cycle checkpoints were activated because cell-cycle progression appeared to be normal in *ecm33*∆ cells. However, further studies are needed to determine how Ecm33 impacts cell-cycle progression.

### 4.4. SAG1 Deletion Perturbs Actin Distribution during Vegetative Growth

*SAG1* (*AGα1*) encodes a cell-adhesion molecule called α-agglutinin in *MATα* cells [42,55], but the function of this molecule during the vegetative growth of *MATa* cells has yet to be identified. In this study, we examined the morphological phenotype of *sag1*∆ *MATa* cells. The results showed that the *sag1*∆ mutation affects the mother and bud cell sizes at the G1 and M stages of the cell cycle, respectively, in *MATa* cells. It also perturbed actin polarization and polarized bud growth. As Sag1 binds directly to Aga1, it would be interesting to investigate the phenotype of *aga1*∆. However, it was difficult to analyze *aga1*∆ because the mutant cells were not suitable for morphological phenotyping due to their propensity to aggregate. The construction of weak alleles of *AGA1* will be necessary to examine the morphological phenotype and investigate its relationship with Sag1.

## 5. Conclusions

This study provided a comprehensive analysis of morphological phenotypes of yeast mannoprotein mutants. The morphology of each cluster of mutants could be explained by the molecular functions of the mannoproteins. The cluster I gene (*CCW12*) encodes a mannoprotein that accounts for 40% of the total mannoproteins in a cell, plays a major structural role, and contributes the most to cell morphogenesis. The cluster II genes (*CCW14*, *FLO11*, *SRL1*, and *TIR3*) do not play structural roles but have similar effects on cell size and cell shape. The cluster V genes (*DSE2*, *EGT2*, and *SUN4*) encode glucosidases, which are required for cell separation. The cluster VI gene (*ECM33*) is required for bud growth and cell wall assembly. Finally, the cluster VII gene (*SAG1*) is required for cell aggregation and is important for determining cell size and actin organization. Cluster III and cluster IV genes do not play major roles in cell morphogenesis. The results presented here increase our understanding of the mechanistic and functional roles of glycoproteins in cell morphogenesis. Morphology-based analysis seems to be a practical means of relating morphological defects to underlying molecular mechanisms, indicating the sensitivity of our approach for determining the responsibility matrix of mannoproteins regarding maintaining the cell wall structure.

## Data Availability

Any additional data will be available upon request to the corresponding author.

## Supplementary Materials

The following are available online at https://www.mdpi.com/article/10.3390/jof7090769/s1, Figure S1. Multivariate analysis of mannoprotein morphological data, Figure S2. Specific morphological features of ccw12Δ cells (cluster I), Figure S3. Specific morphological features of ccw12Δ buds (cluster I), Figure S4. Complete gel of western blotting of phosphorylated Slt2, Figure S5. Specific morphological features of mother cells of all mutants in cluster II, Figure S6. Specific morphological features shared among members of cluster V, Figure S7. Morphological defects in cluster V do not affect the cell cycle, Figure S8. Specific morphological features of ecm33Δ (cluster VI), Figure S9. Morphological parameters related to cell size in ecm33Δ (cluster VI) versus other mannoprotein mutants, Figure S10. Specific morphological features of sag1Δ (cluster VII), Figure S11. Specific actin-related morphological features of sag1Δ (cluster VII) in other mutants, Figure S12. Gene homology among mannoproteins, Figure S13. Morphological similarity of mannoproteins and drug-treated wild-type cells, Figure S14. Protein–protein interaction (PPIs) network, Figure S15. Genetic interaction network, Figure S16. Chemical-genetic profile of mannoproteins, Table S1. List of 36 cell wall mannoprotein strains, Table S2. List of strains to study kinetics of mannoproteins (Dse2 and Egt2; cluster V) and proteins involved in the cytokinesis and cell separation, Table S3. Oligonucleotides (A) and plasmids (B) used in this study, Table S4. Parameters showed statistically significant difference at least in one mutant compared with the null distribution (Wald test, FDR = 0.05), Table S5. Significant loadings of first five PC spaces (used for GMM clustering) after Bonferroni correction (*t*-test, *p* < 0.05), Table S6. Significant parameters of each mannoprotein mutant (Wald test, FDR = 0.05), Table S7. Pearson correlation coefficient (r)1 between Dse2-GFP and other proteins, Table S8. Duplicated and non-duplicated genes of mannoproteins considering their functional responsibility and diversification, Table S9. Protein-Protein interactions of 32 mannoproteins. Data obtained from Oughtred et al. (2019), Table S10. Genetic interactions of 32 mannoproteins. Data obtained from Costanzo et al. (2016), Table S11. Chemical-genetic profile of 32 mannoprotein mutants. Data obtained from the *Saccharomyces* Genome Database (SGD).
