# Supplementary material for: Defining Functions of Mannoproteins in Saccharomyces cerevisiae by High-Dimensional Morphological Phenotyping
Source: J Fungi (Basel). 2021 Sep 17;7(9):769. doi: 10.3390/jof7090769 (PMC8466635; doi:10.3390/jof7090769)
Supplement: Supplementary file 1 [file jof-07-00769-s001.zip › Supplementary Figures.pdf]

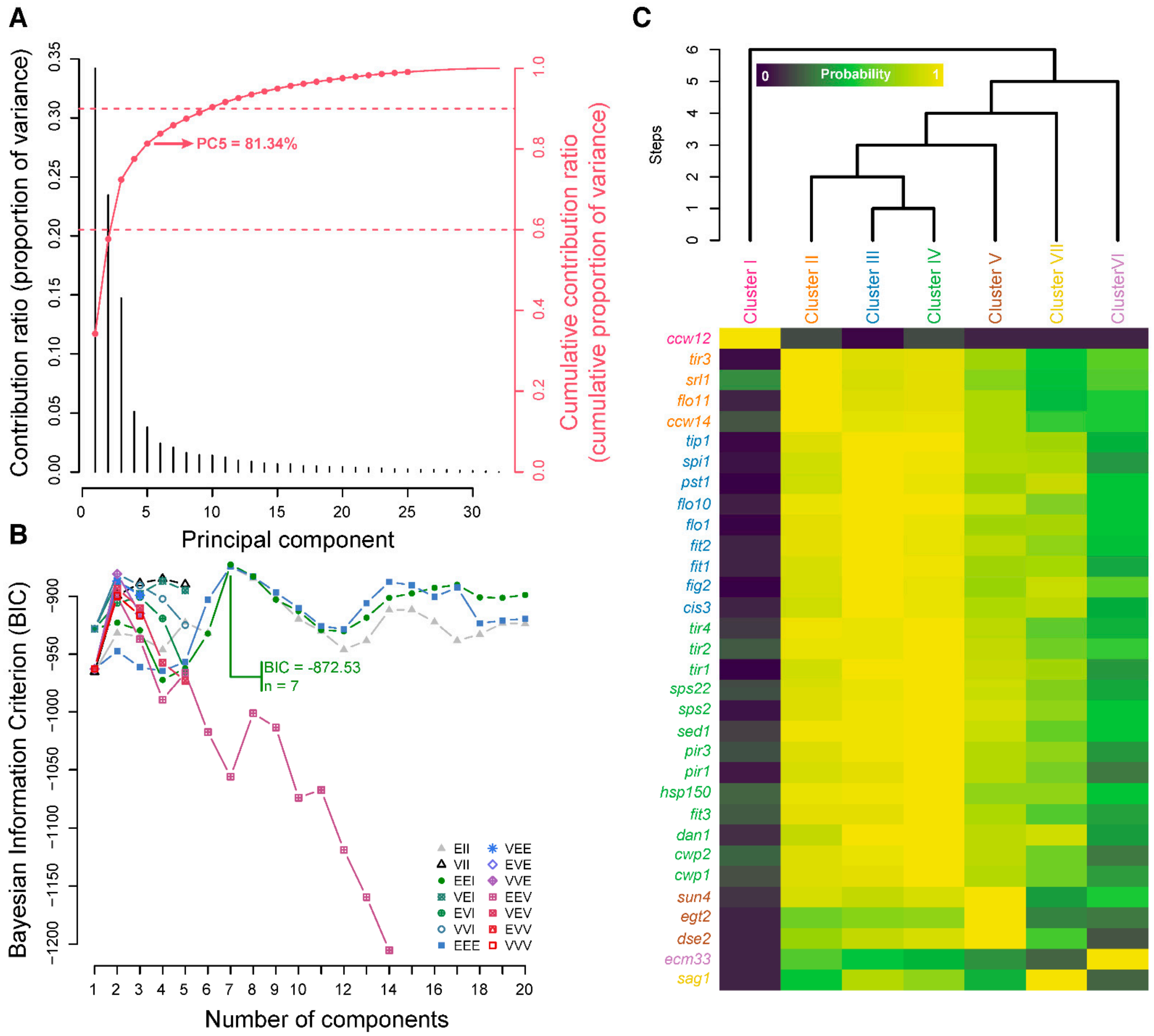

Figure S1. Multivariate analysis of mannoprotein morphological data. (**A**). Principal component (PC) analysis. Black bars (left axis) indicate the contribution ratio (proportion of variance), red circles (right axis) indicate the cumulative proportion of variance (cumulative contribution ratio; CCR), and horizontal dashed lines (right axis) indicate CCRs of 60% and 90%. The first five PC scores were used for Gaussian mixture model (GMM) clustering (see Figure 1). (**B**). Defining the number of components for GMM clustering. The number of components describing the underlying Gaussian distributions was defined based on Bayesian information criterion values of models with differing parametrizations. Using the first five PC scores, an EEI model (diagonal distributions with equal volume and shape as well as coordinate axes) with seven components provided the best fit. (**C**). Posterior group membership probability matrix. Each value shows the posterior probability based on the EEI model in "**B**". The posterior probability describes the likelihood of each mutant belonging to each cluster. The highest values were used to assign members to each cluster. Logarithmic transformation of conditional probabilities from expectation maximization was used to generate the heatmap. The dendrogram illustrates model-based hierarchical agglomerative clustering based on the Gaussian probability model for maximizing the resulting likelihood. Mutants are color-coded according to GMM clustering of morphological data (see Figure 1).

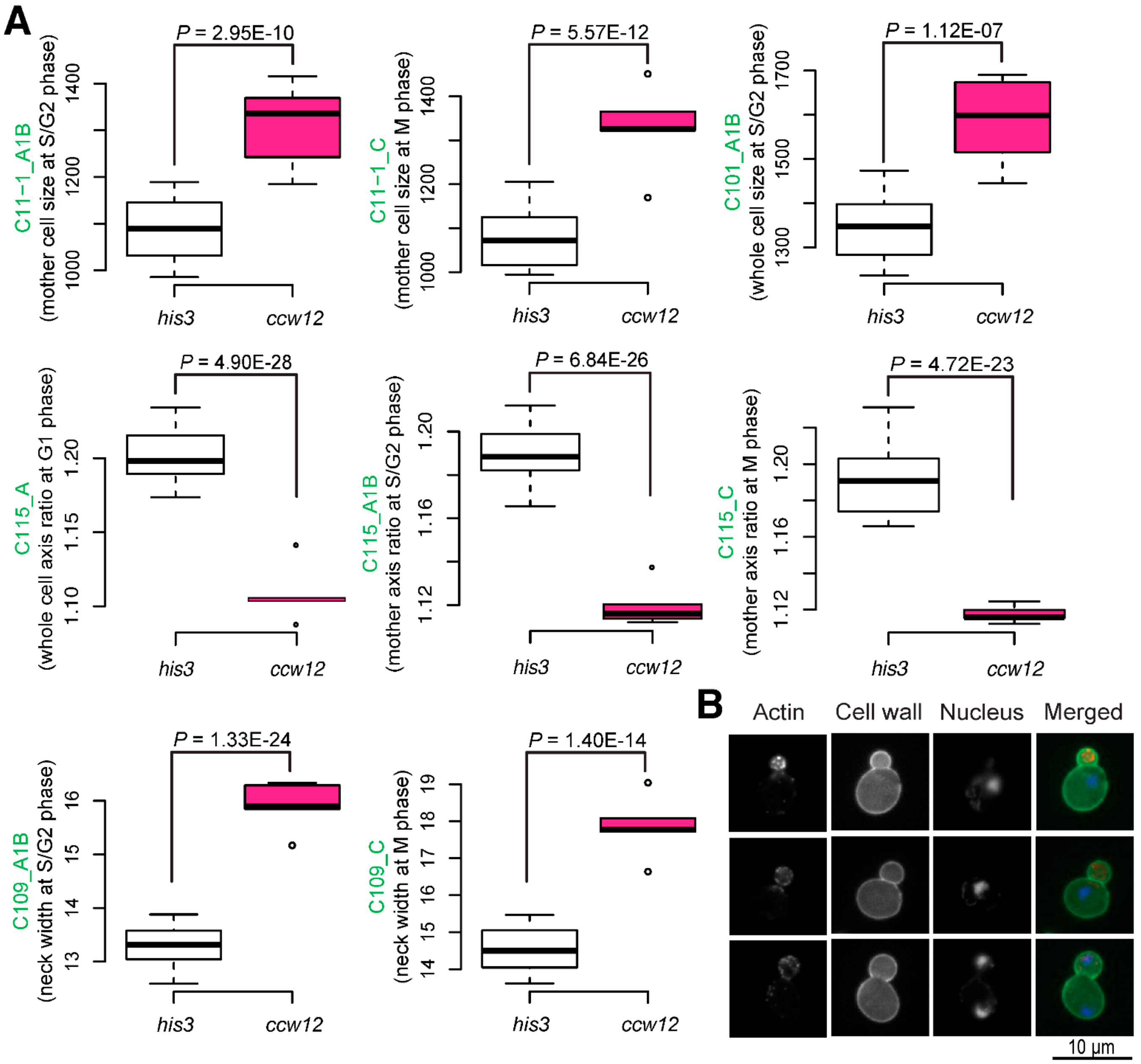

Figure S2. Specific morphological features of *ccw12*Δ cells (cluster I). (**A**). Box plots of parameters related to cell size (C11-1_A1B, C11-1_C, and C101_A1B), mother cell shape (C115), and neck width (C109) in *ccw12*Δ cells in comparison with wild-type (WT) cells (*his3*). *p*-values from the Wald-test are also shown. (**B**). Examples of triple-stained *ccw12*Δ cells (red, green, and blue for actin, the cell wall, and the nucleus, respectively) are shown. The area unit is the number of pixels squared, the ratio parameters are unitless, and the unit for length is the number of pixels; for details, see the CalMorph user at http://www.yeast.ib.k.u-tokyo.ac.jp/CalMorph/download.php?path=CalMorph-manual.pdf.

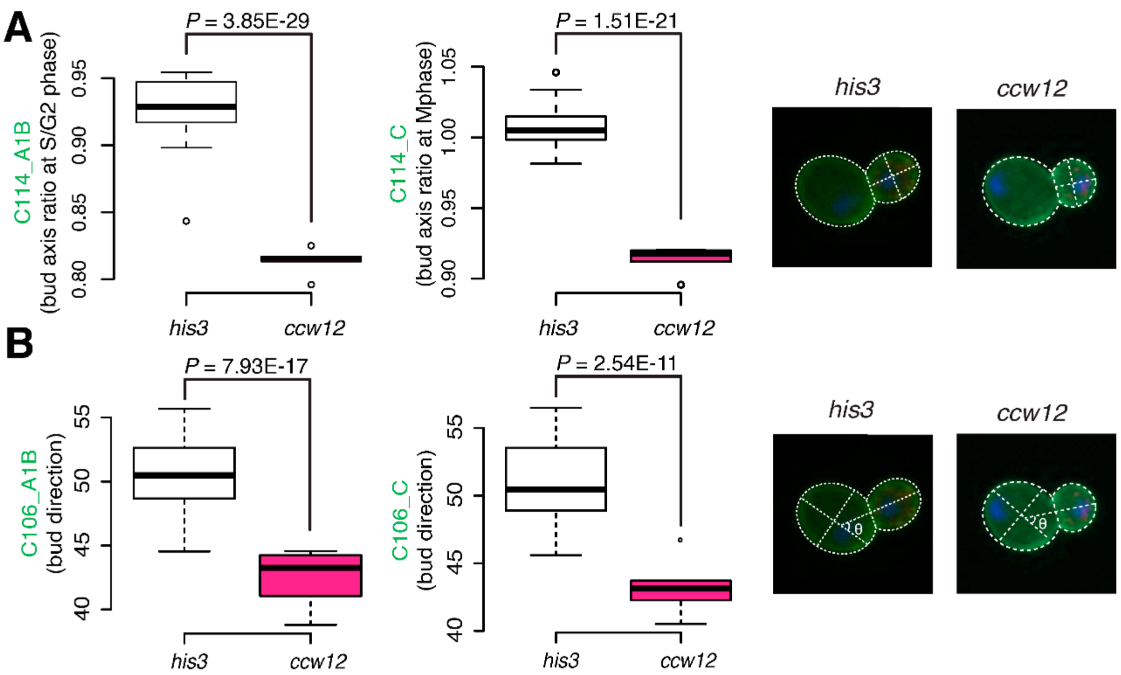

Figure S3. Specific morphological features of *ccw12*Δ buds (cluster I). Box plots of parameters related to bud shape (C114; **A**) and bud direction (C106; **B**) in *ccw12*Δ cells in comparison with WT cells (*his3*). *p*-values from the Wald-test are also shown. Examples of *ccw12*Δ cells triple-stained (red, green, and blue for actin, the cell wall, and the nucleus, respectively) to determine the bud shape (top: ratio of bud long axis to bud short axis) and bud direction (bottom: θ is the angle between the extension of the line from the tip of the bud to the midpoint of the neck and the mother cell long axis) are shown. Ratio parameters are unitless; for details, see the CalMorph user at http://www.yeast.ib.k.u-tokyo.ac.jp/CalMorph/download.php?path=CalMorph-manual.pdf.

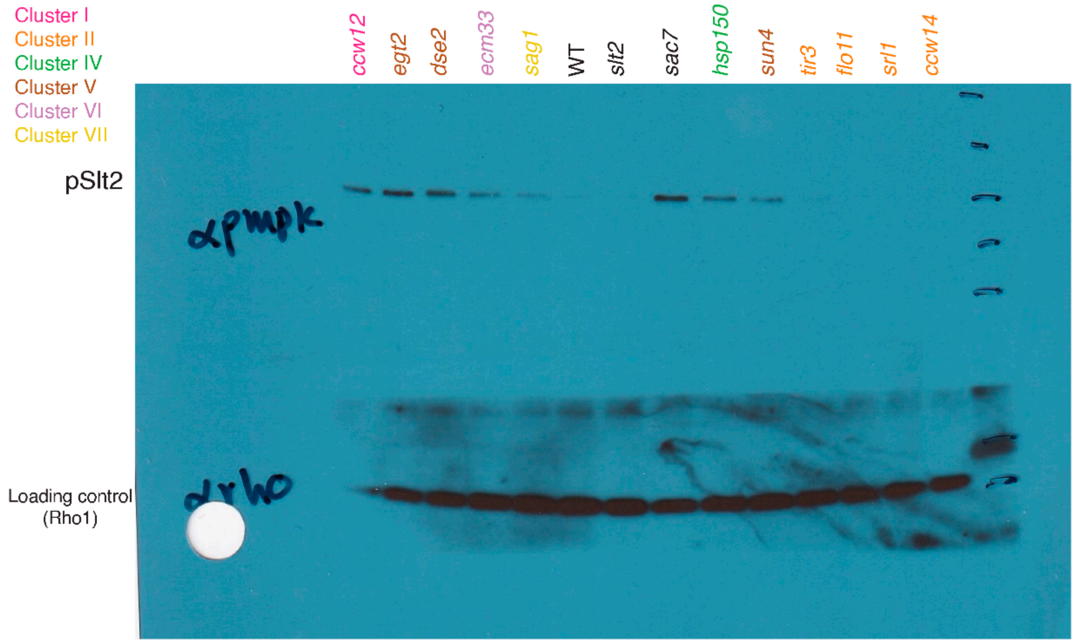

Figure S4. Complete gel of western blotting of phosphorylated Slt2. BY4743 (WT); *ccw12*Δ/*ccw12*Δ (cluster I); *ccw14*Δ/*ccw14*Δ, *flo11*Δ/*flo11*Δ, *srl1*Δ/*srl1*Δ and *tir3*Δ/*tir3*Δ (cluster II); *hsp150*Δ/*hsp150*Δ (cluster IV); *dse2*Δ/*dse2*Δ, *egt2*Δ/*egt2*Δ, and *sun4*Δ/*sun4*Δ (cluster V); *ecm33*Δ/*ecm33*Δ (cluster VI); and *sag1*Δ/*sag1*Δ (cluster VII) cells were examined for the presence of phosphorylated Slt2. Rabbit antibody against phospho-p42/44 MAPK (T202/Y204) and rabbit antibody against yeast Rho1 were used to detect the phosphorylated Slt2 and Rho1, respectively. *slt2*Δ/*slt2*Δ and *sac7*Δ/*sac7*Δ were used as negative and positive controls, respectively, for phosphorylated Slt2. Mutants are color-coded according to Gaussian mixture model clustering of morphological data (see Figure 1).

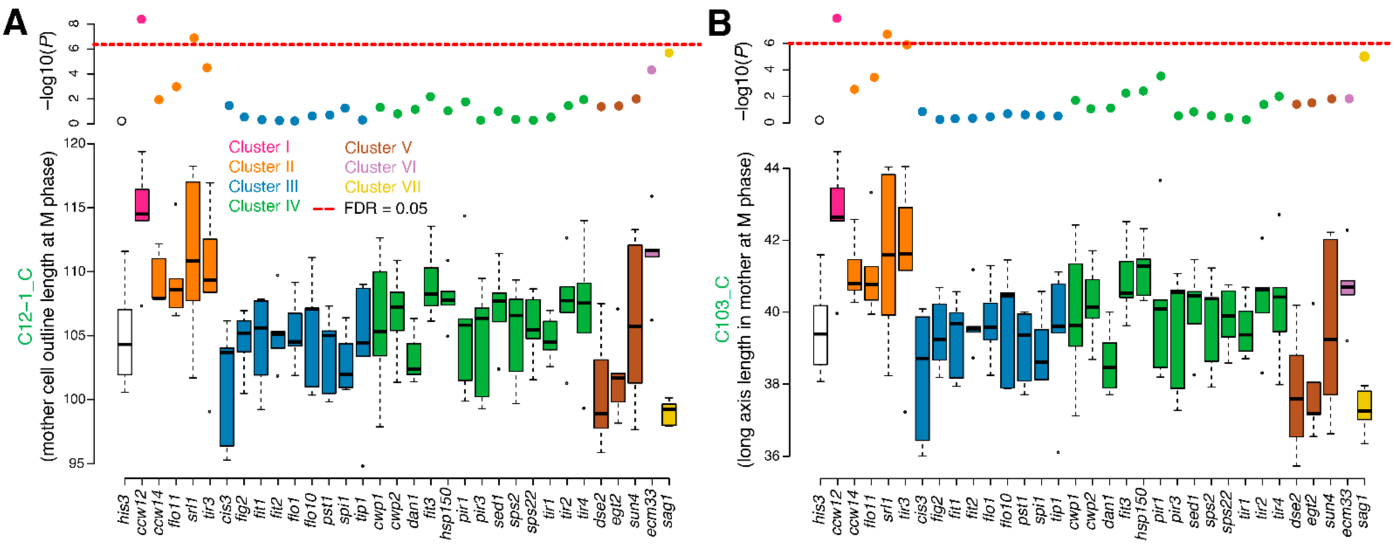

Figure S5. Specific morphological features of mother cells of all mutants in cluster II. Box plots of mother cell size (C12-1_C; **A**) and long axis length (C103_C; **B**) at the M phase in WT cells (*his3*) and 32 mannoprotein mutants. Scatter plots include $-\log_{10}$ of *p*-values from the Wald-test. Mutants are color-coded according to GMM clustering of morphological data (see Figure 1). The unit for length is the number of pixels; for details, see the CalMorph user at http://www.yeast.ib.k.u-tokyo.ac.jp/CalMorph/download.php?path=CalMorph-manual.pdf.

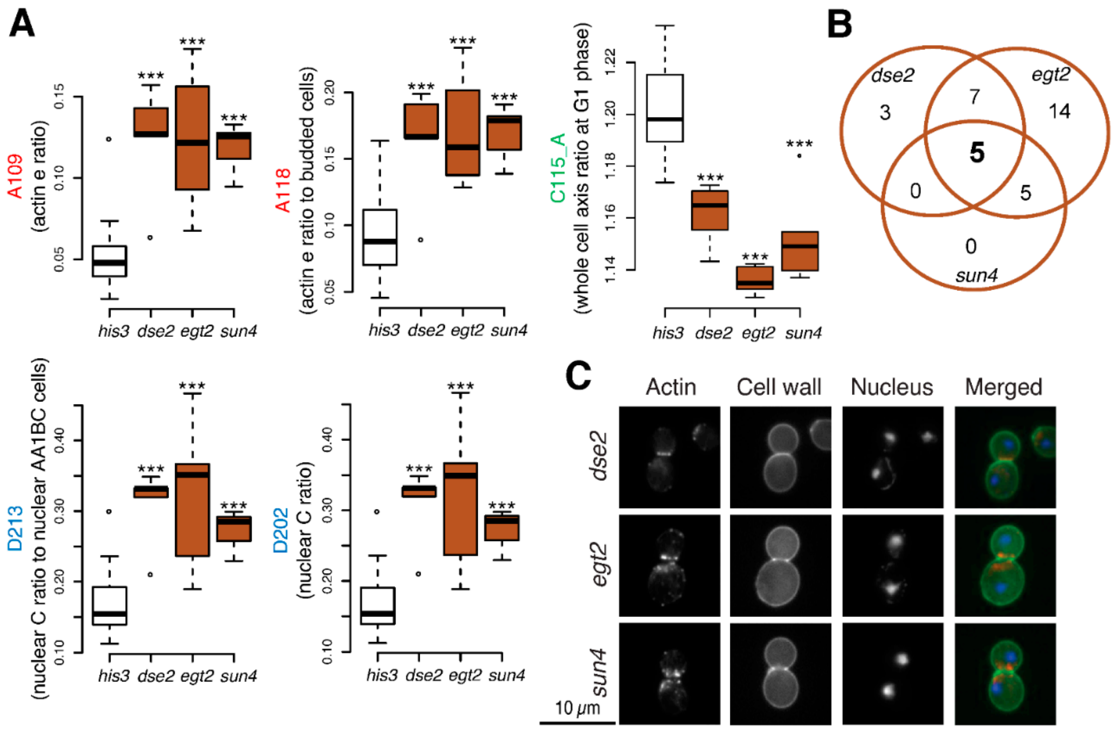

Figure S6. Specific morphological features shared among members of cluster V. (**A**). Box plots of five morphological defects observed in all members of cluster V (*dse2*Δ, *egt2*Δ, and *sun4*Δ) in comparison with WT cells (*his3*). Parameters are color-coded with those related to actin, the cell wall, and the nucleus shown in red, green, and blue, respectively. *** $p <$ 1E−06 (Wald-test). Ratio parameters are unitless; for details, see the CalMorph user at http://www.yeast.ib.k.u-tokyo.ac.jp/CalMorph/download.php?path=CalMorph-manual.pdf. For more details on A109, A118, and D213, see Supporting Figure 5 in [14]. (**B**). Venn diagram of parameters with significant changes in *dse2*Δ (n = 15), *egt2*Δ (n = 31), and *sun4*Δ (n = 10), also see Table S6. (**C**). Examples of triple-stained unseparated mother/daughter cells (red, green, and blue staining indicates actin, the cell wall, and the nucleus, respectively) for each mutant.

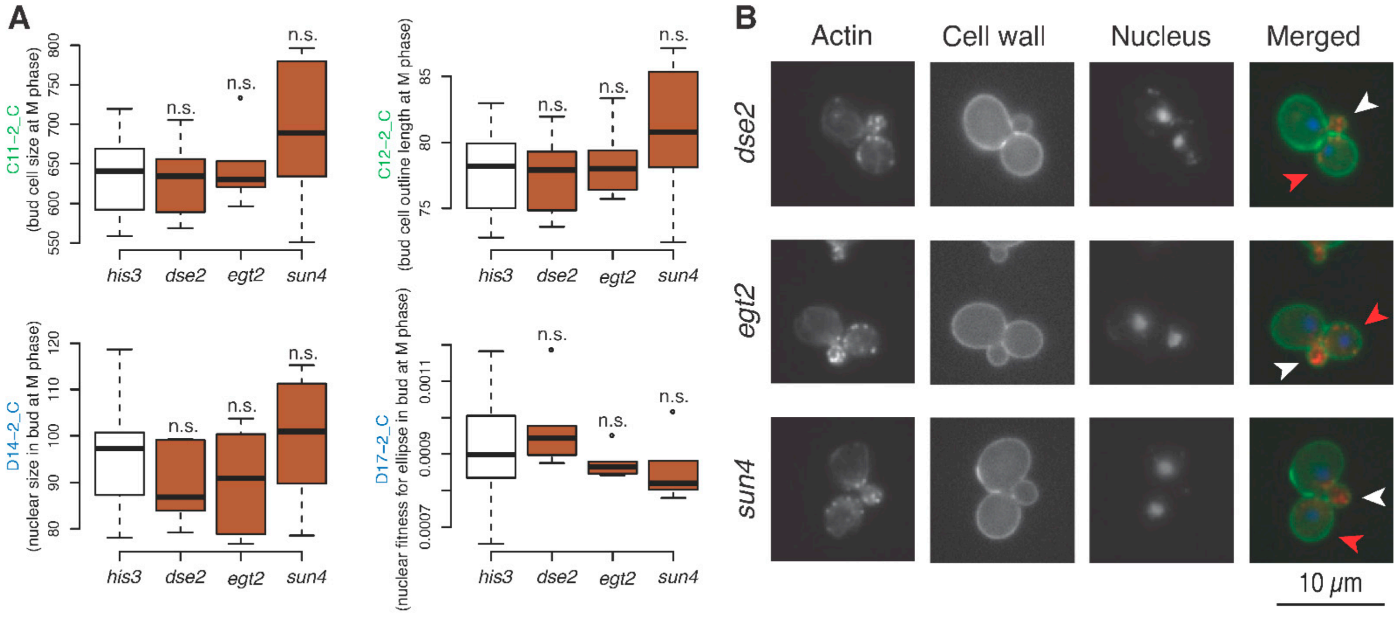

Figure S7. Morphological defects in cluster V do not affect the cell cycle. (**A**). Box plots of bud cell size (C11-2_C and C12-2_C) and bud nuclear size (D14-2_C and D17-2_C) at the M phase in members of cluster V (*dse2*Δ, *egt2*Δ, and *sun4*Δ) in comparison with WT cells (*his3*). n.s., not significant (FDR = 0.05, Wald-test). The area unit is the number of pixels squared, and the unit for length is the number of pixels; for details, see the CalMorph user at http://www.yeast.ib.k.u-tokyo.ac.jp/CalMorph/download.php?path=CalMorph-manual.pdf. (**B**). Examples of triple-stained cells (red, green, and blue staining indicates actin, the cell wall, and the nucleus, respectively) of members of cluster V for whom mother cells have two buds. White arrows indicate new buds occurring before the complete separation of mother/daughter cells (red arrows) from the previous cell cycle, implying successful progression of the cell cycle.

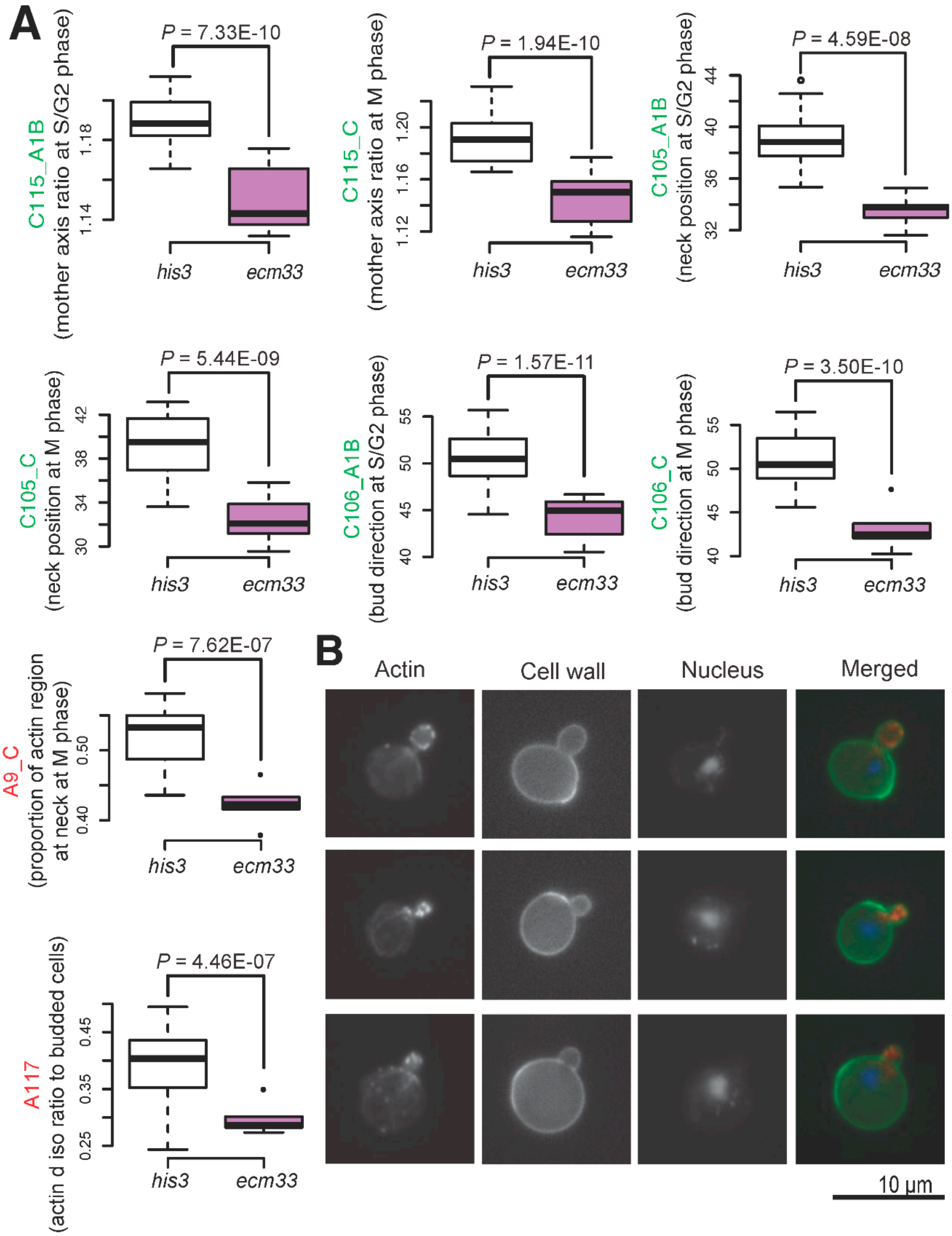

Figure S8. Specific morphological features of *ecm33*Δ (cluster VI). (**A**). Box plots of parameters related to mother cell shape (C115), neck position (C105), and bud direction (C106) at S/G2 and M phases as well as actin distribution (for more details on A9_C and A117, see Supporting Figure 5 of [14]) in *ecm33*Δ cells in comparison with WT cells (*his3*). *p*-values from the Wald-test are also shown. Ratio parameters are unitless; for details, see the CalMorph user at http://www.yeast.ib.k.u-tokyo.ac.jp/CalMorph/download.php?path=CalMorph-manual.pdf. (**B**). Examples of triple-stained *ecm33*Δ cells (red, green, and blue staining indicates actin, the cell wall, and the nucleus, respectively) are shown.

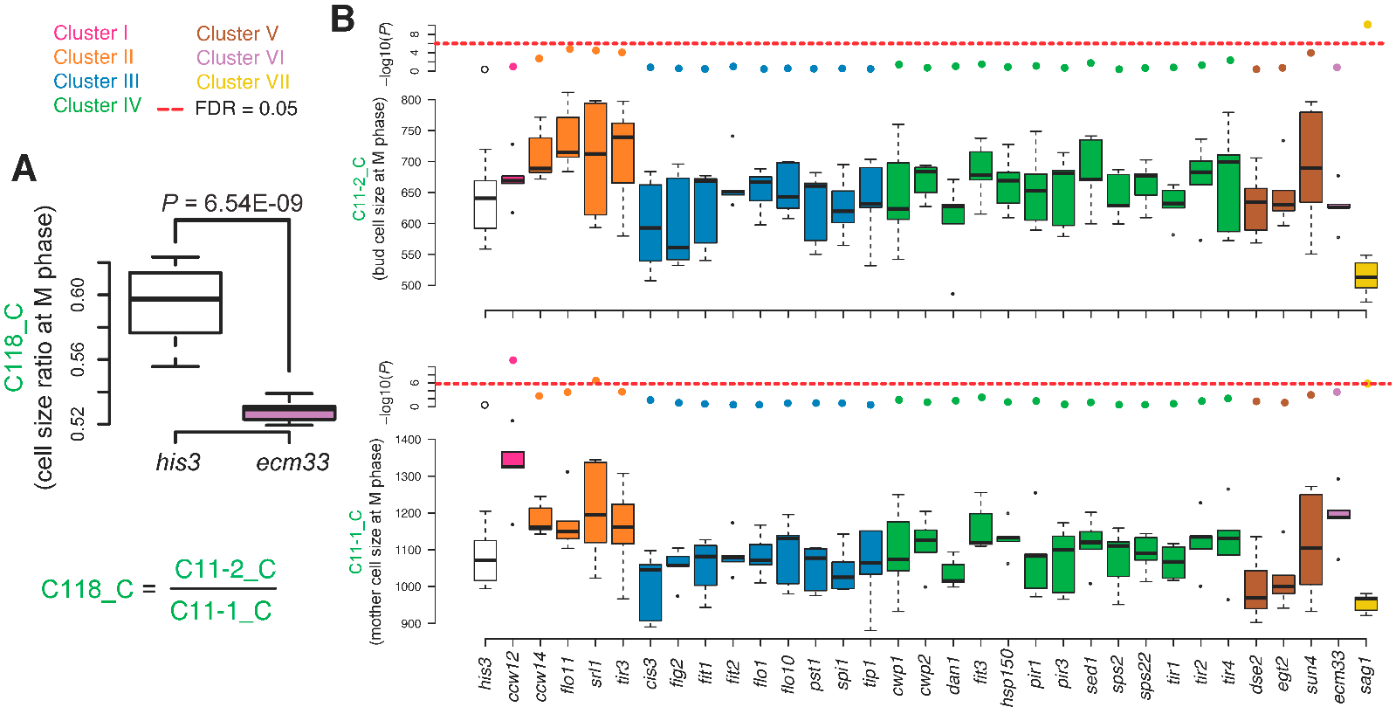

Figure S9. Morphological parameters related to cell size in *ecm33*Δ (cluster VI) versus other mannoprotein mutants. (**A**). Boxplot of the cell size ratio (118_C) in *ecm33*Δ cells compared with WT cells (*his3*). The cell size ratio is the ratio of bud cell size (C11-2_C) to mother cell size (C11-1_C). *p*-value from the Wald-test is also shown. The ratio parameters are unitless. (**B**). Box plots of WT cells (*his3*) and 32 mannoprotein mutants. Scatter plots include $-\log_{10}$ of *p*-values from the Wald-test. Mutants are color-coded according to the results of GMM clustering of morphological data (see Figure 1). The unit for size is the number of pixels squared; for details, see the CalMorph user at http://www.yeast.ib.k.u-tokyo.ac.jp/CalMorph/download.php?path=CalMorph-manual.pdf.

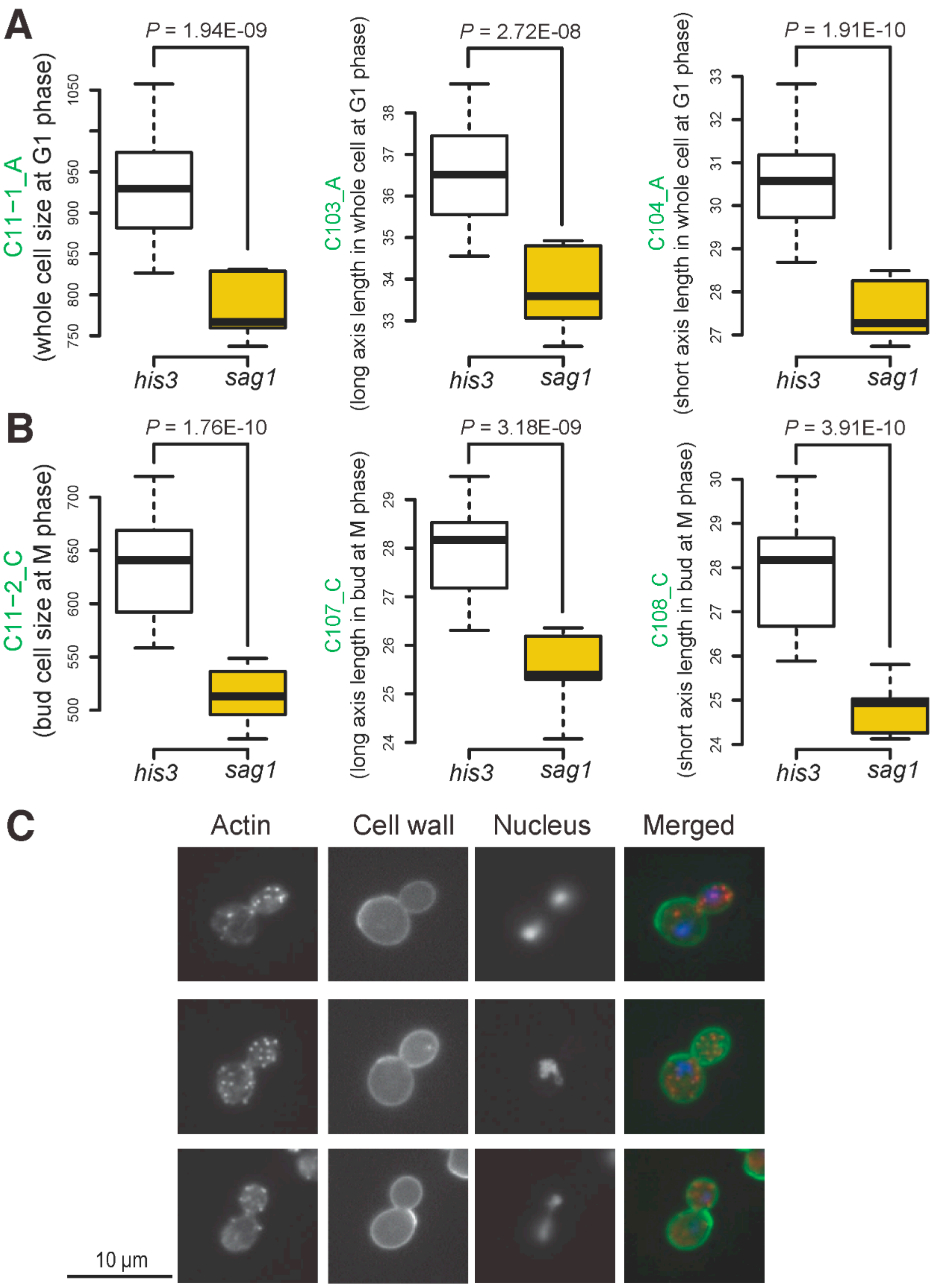

Figure S10. Specific morphological features of *sag1*Δ (cluster VII). (**A**). Box plots of parameters related to cell size (C11-1_A) and mother cell axis length (C103_A, and C104_A) in *sag1*Δ cells in comparison with WT cells (*his3*). (**B**). Box plots of parameters related to bud cell size (C11-2_C) and bud axis length (C107_C and C108_C) in *sag1*Δ cells in comparison with WT cells (*his3*). *p*-values from the Wald-test are also shown. (**C**). Examples of triple-stained *sag1*Δ cells (red, green, and blue staining indicates actin, the cell wall, and the nucleus, respectively). The units for area and length are the number of pixels squared and the number of pixels, respectively; for details, see the CalMorph user at http://www.yeast.ib.k.u-tokyo.ac.jp/CalMorph/download.php?path=CalMorph-manual.pdf.

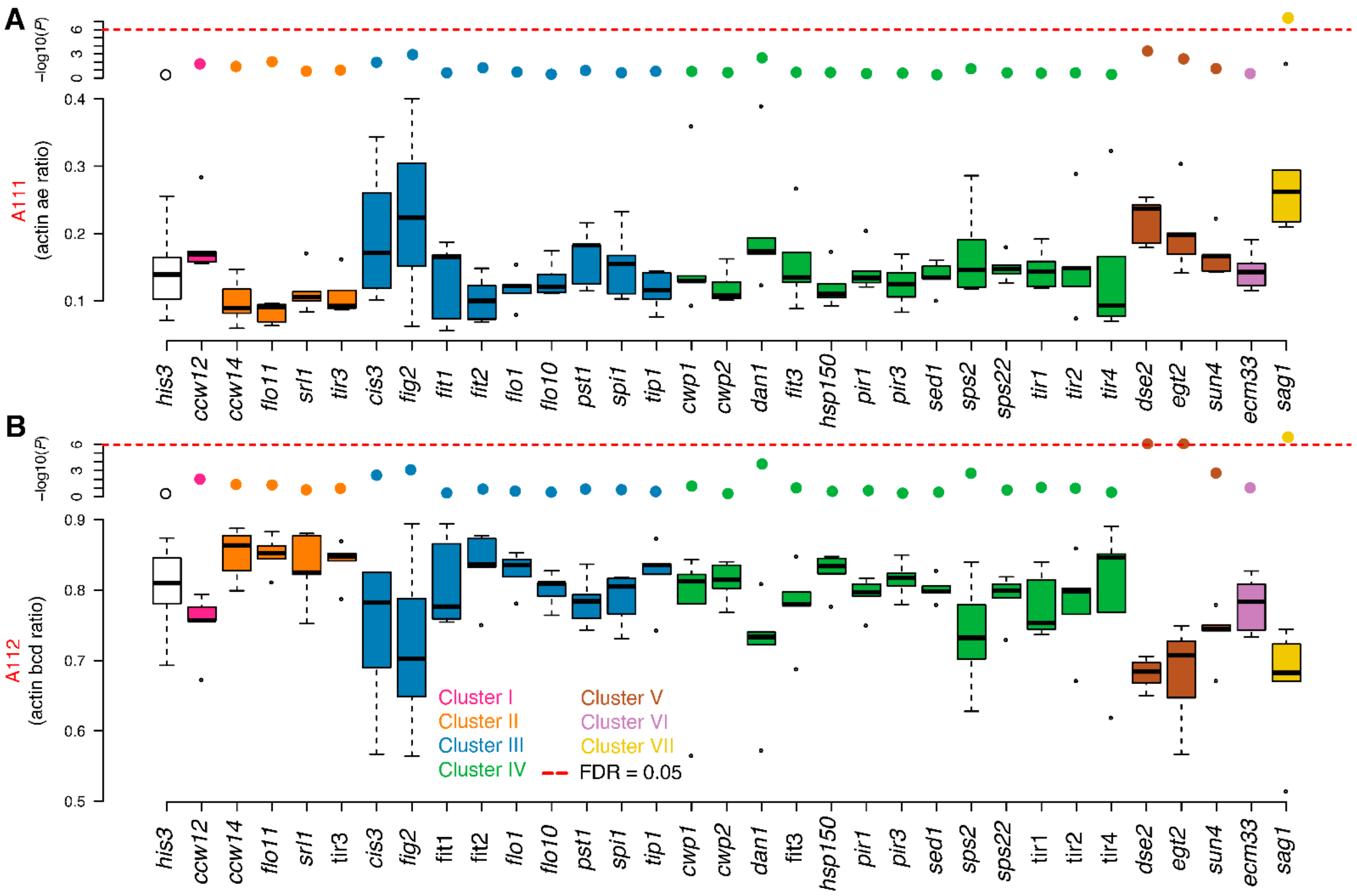

Figure S11. Specific actin-related morphological features of *sag1*Δ (cluster VII) in other mutants. Box plots of A111 (**A**) and A112 (**B**), parameters related to actin ratios, across all 32 mannoprotein mutants in comparison with WT cells (*his3*). For more details on A111 and A112, see Supporting Figure 5 of [14]. Scatter plots include $-\log_{10}$ of p-values from the Wald-test. Mutants are color-coded according to GMM clustering of morphological data (see Figure 1). The ratio parameters are unitless; for details, see the CalMorph user at http://www.yeast.ib.k.u-tokyo.ac.jp/CalMorph/download.php?path=CalMorph-manual.pdf.

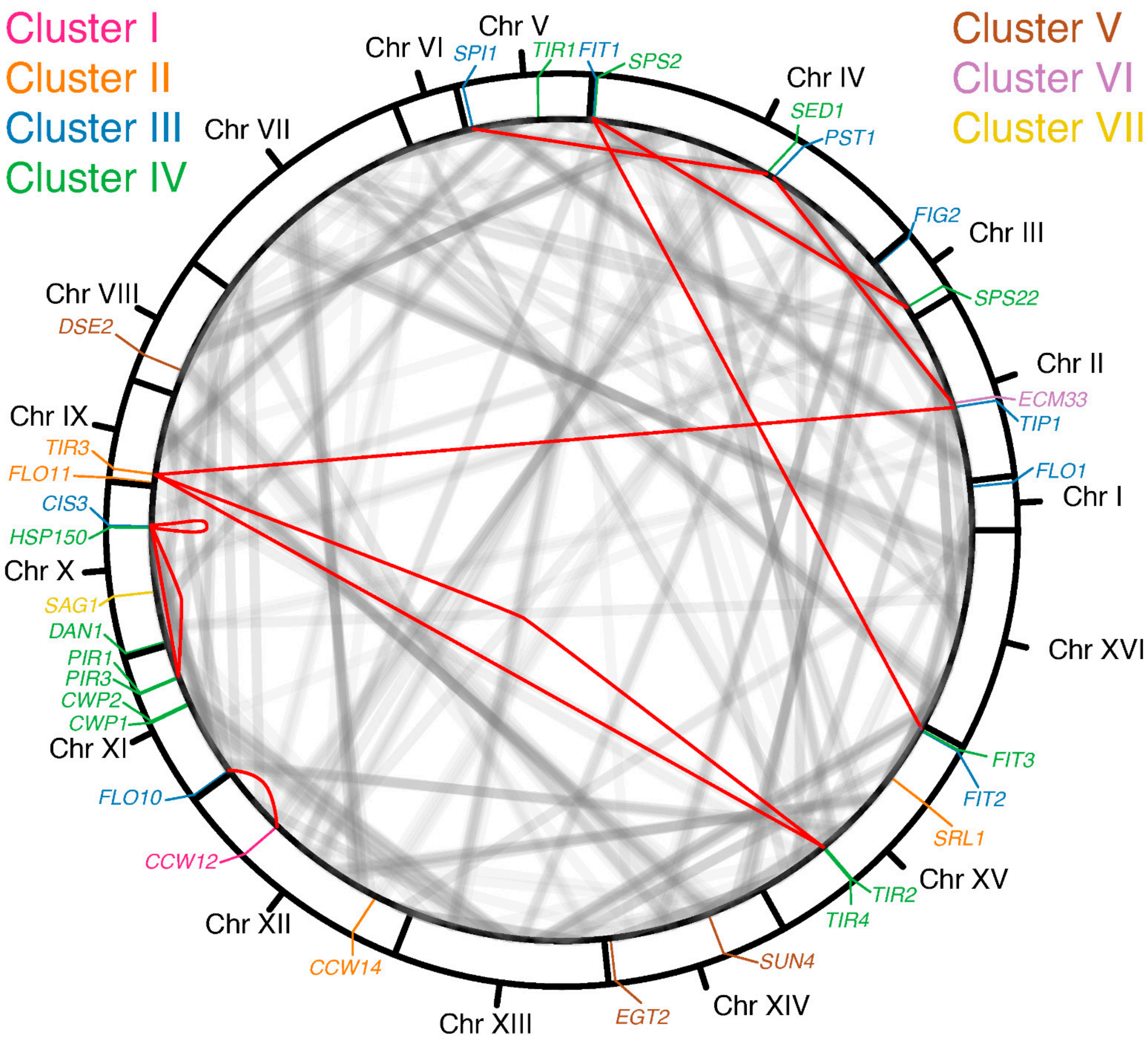

Figure S12. Gene homology among mannoproteins. Gray lines indicate gene homology among all yeast genes. Data were obtained from the Yeast Gene Order Browser [56]. Red lines connect paralogs among 32 mannoproteins (Table S1). Mannoproteins are color-coded according to GMM clustering of morphological data (see Figure 1).

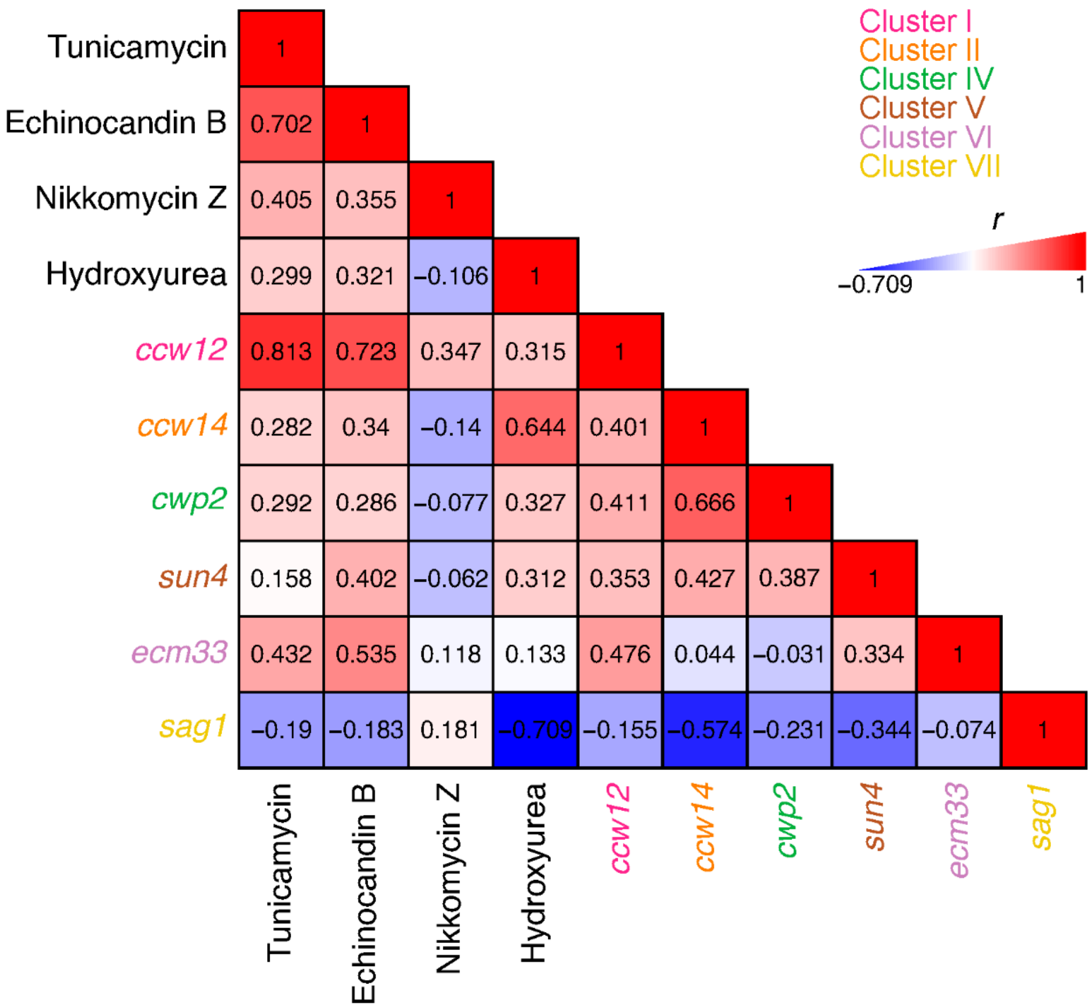

Figure S13. Morphological similarity of mannoproteins and drug-treated wild-type cells. Morphological similarity of *ccw12*Δ (I), *ccw14*Δ (II), *cwp2*Δ (IV), *sun4*Δ (V), *ecm33*Δ (VI), and *sag1*Δ (VII) with tunicamycin (affecting protein glycosylation), echinocandin B (affecting 1,3-β-glucan synthesis), nikkomycin Z (affecting chitin synthesis), and hydroxyurea (affecting DNA replication). Numbers incidate Pearson correlation coefficient (*r*). Mannoproteins are color-coded according to GMM clustering of morphological data (see Figure 1).

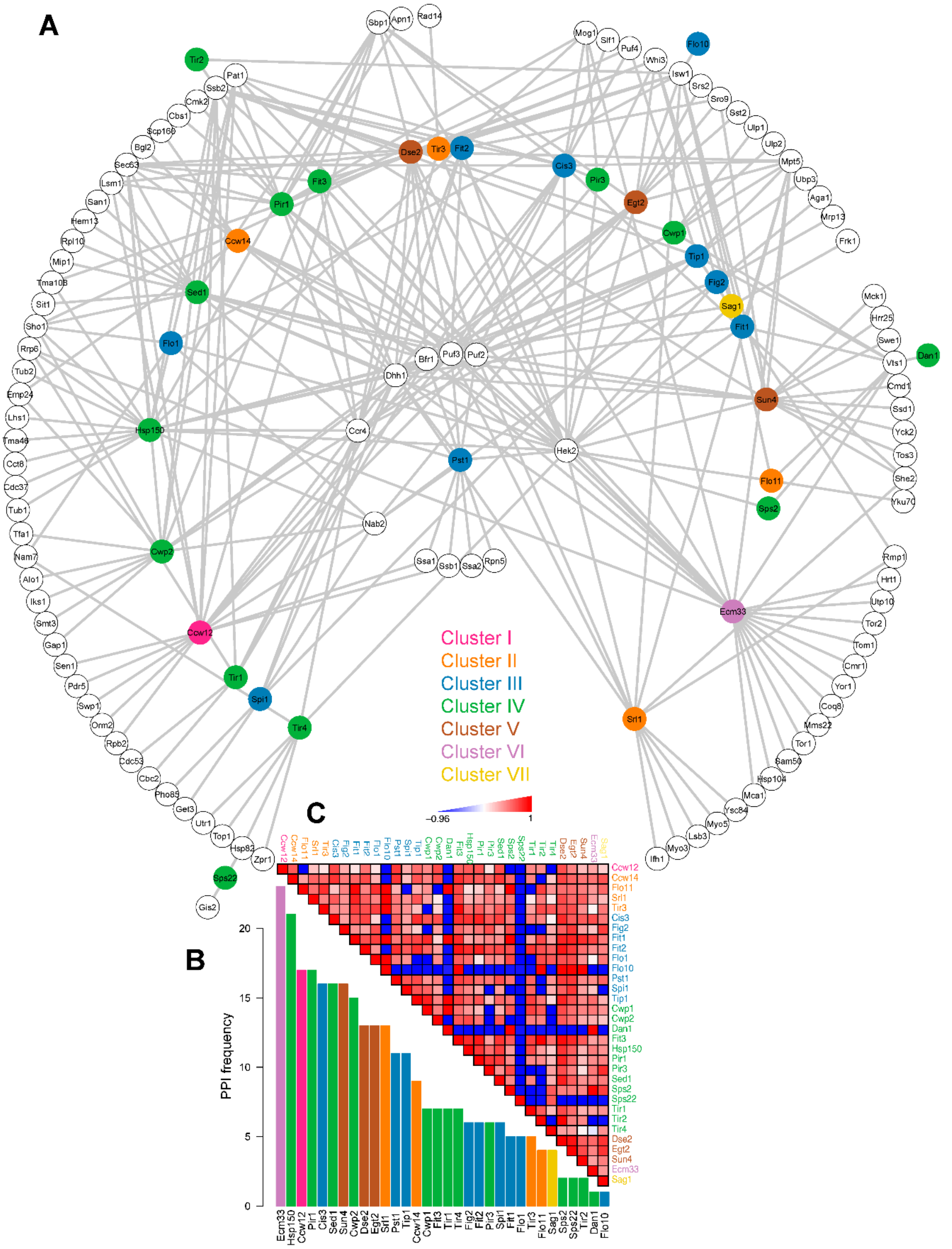

Figure S14. Protein–protein interaction (PPIs) network. (**A**). Protein–protein interactome profile of 32 mannoproteins (colored circles). White circles represent various proteins in the cell proteome (Table S9). Data were obtained from the BioGRID database [36]. (**B**). Bar plot showing the frequencies of PPIs. (**C**). Degrees of association among 32 mannoproteins according to the interactome profile based on tetrachoric correlation (ρ) given the PPI network in "**A**". In all sections, mannoproteins are color-coded according to GMM clustering of morphological data (see Figure 1).

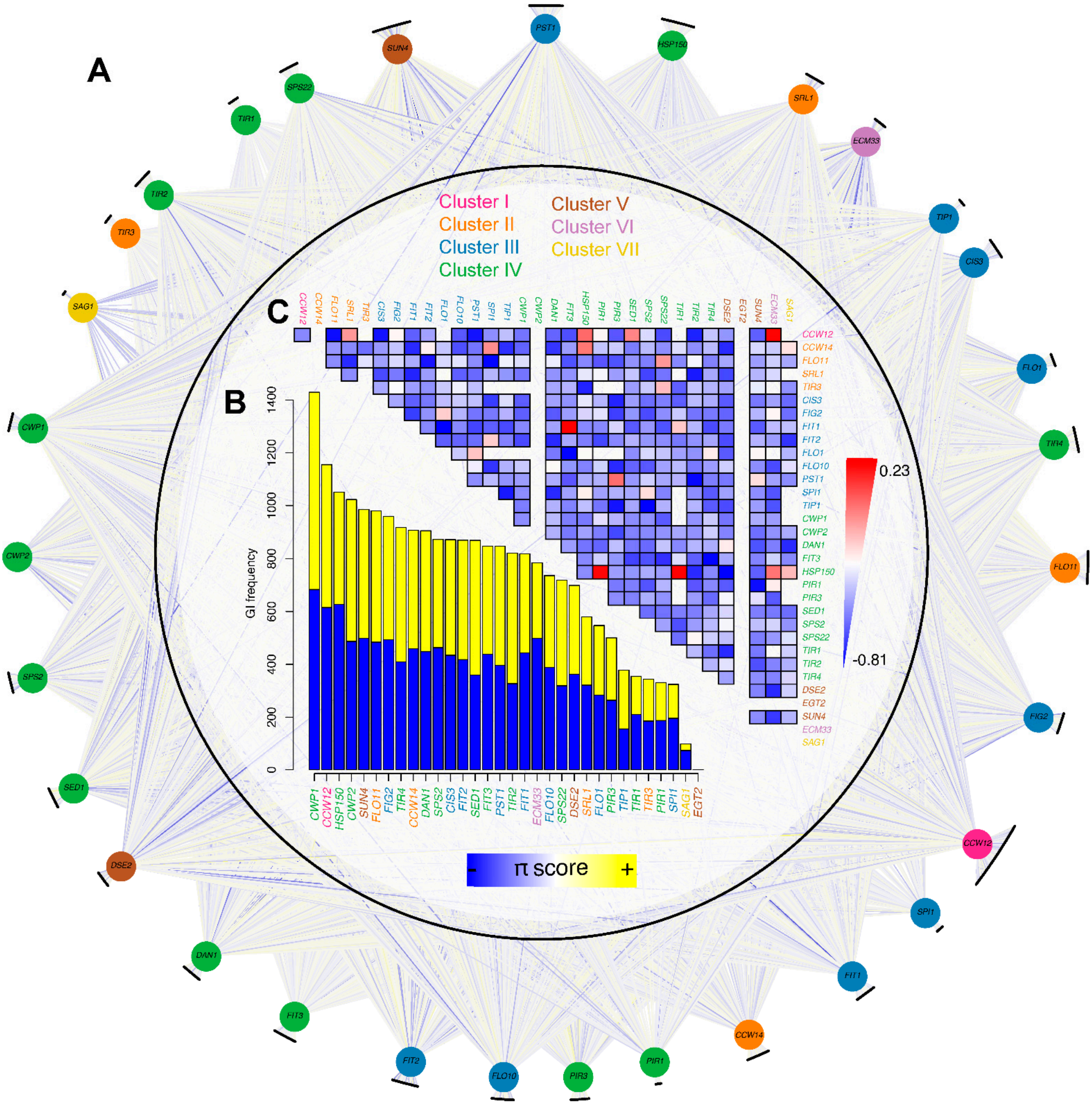

Figure S15. Genetic interaction network. (**A**). Genetic interactome profile of 31 mannoproteins (colored circles) with blue and yellow indicating significant negative and positive interactions ($p < 0.05$). *EGT2* was not found to have any significant interactions. Black circles represent different genes in the cell (Table S10). Data were obtained from [38]. (**B**). Bar plot showing frequencies of genetic interactions. (**C**). Genetic interaction profile similarity among 32 mannoproteins according to the interactome profile obtained from Data file S3 in [38]. In all sections, mannoproteins are color-coded according to GMM clustering of morphological data (see Figure 1).

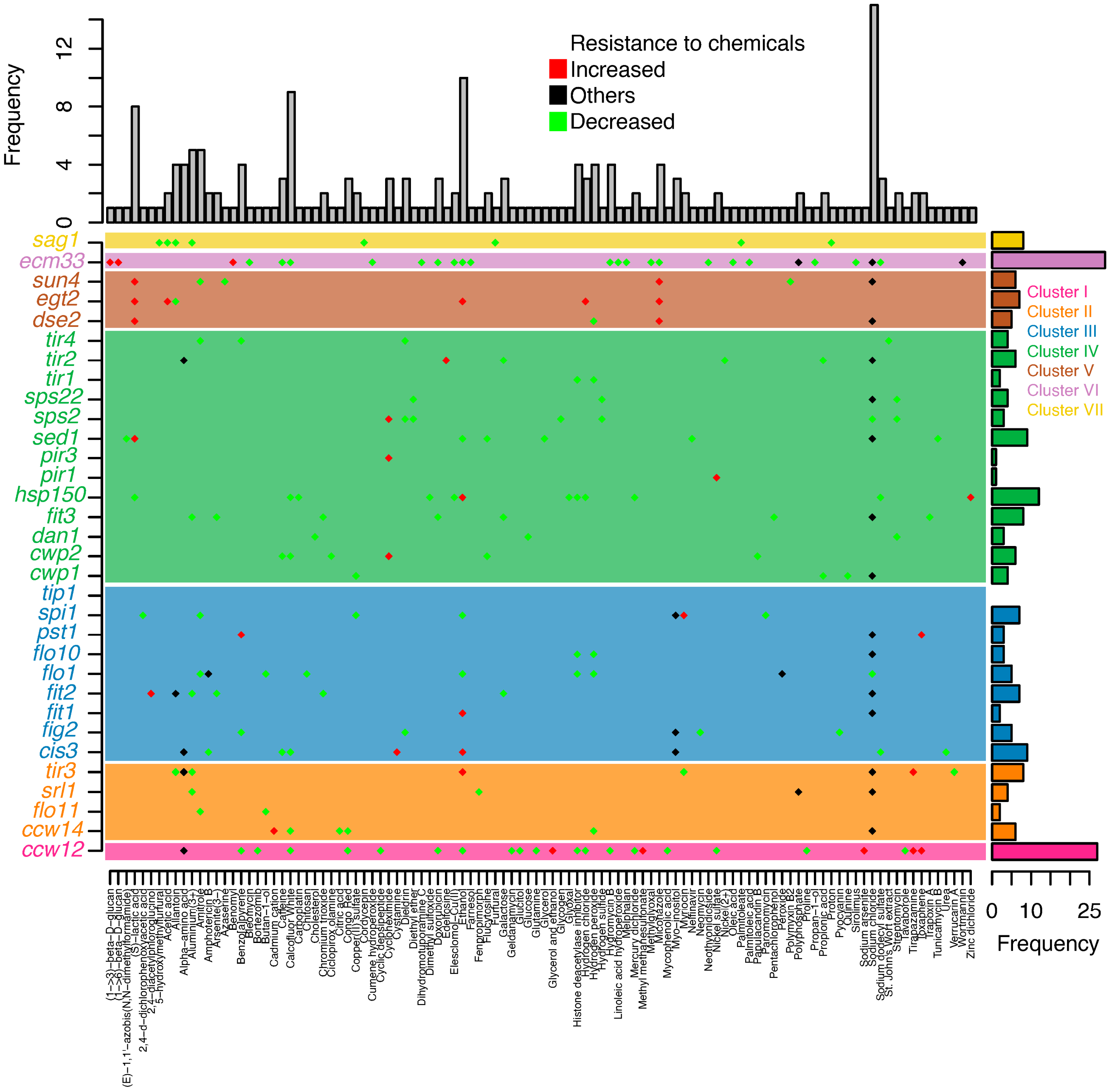

Figure S16. Chemical-Genetic profile of mannoproteins. Scatter plot of the phenotype responses of 32 mannoproteins mutants based on 106 components (Table S11). Data were obtained from the *Saccharomyces* Genome Database. The gray bar plot shows the frequencies of the components tested across all mutants. The horizontal bar plot shows the frequency of each mannoprotein mutant in all experiments. Mannoproteins are color-coded according to GMM clustering of morphological data (see Figure 1).
